# Mitochondrial heteroplasmy and DNA barcoding in Hawaiian *Hylaeus *(*Nesoprosopis*) bees (Hymenoptera: Colletidae)

**DOI:** 10.1186/1471-2148-10-174

**Published:** 2010-06-11

**Authors:** Karl N Magnacca, Mark JF Brown

**Affiliations:** 1 Department of Zoology, School of Natural Sciences, Trinity College Dublin, Dublin 2, Ireland; 2 Current address: Department of Biology, University of Hawai'i, 200 W. Kawili St., Hilo HI 96720, USA; 3 School of Biological Sciences, Royal Holloway University of London, Egham, Surrey, TW20 0EX, UK

## Abstract

**Background:**

The past several years have seen a flurry of papers seeking to clarify the utility and limits of DNA barcoding, particularly in areas such as species discovery and paralogy due to nuclear pseudogenes. Heteroplasmy, the coexistence of multiple mitochondrial haplotypes in a single organism, has been cited as a potentially serious problem for DNA barcoding but its effect on identification accuracy has not been tested. In addition, few studies of barcoding have tested a large group of closely-related species with a well-established morphological taxonomy. In this study we examine both of these issues, by densely sampling the Hawaiian *Hylaeus *bee radiation.

**Results:**

Individuals from 21 of the 49 *a priori *morphologically-defined species exhibited coding sequence heteroplasmy at levels of 1-6% or more. All homoplasmic species were successfully identified by COI using standard methods of analysis, but only 71% of heteroplasmic species. The success rate in identifying heteroplasmic species was increased to 86% by treating polymorphisms as character states rather than ambiguities. Nuclear pseudogenes (numts) were also present in four species, and were distinguishable from heteroplasmic sequences by patterns of nucleotide and amino acid change.

**Conclusions:**

Heteroplasmy significantly decreased the reliability of species identification. In addition, the practical issue of dealing with large numbers of polymorphisms- and resulting increased time and labor required - makes the development of DNA barcode databases considerably more complex than has previously been suggested. The impact of heteroplasmy on the utility of DNA barcoding as a bulk specimen identification tool will depend upon its frequency across populations, which remains unknown. However, DNA barcoding is still likely to remain an important identification tool for those species that are difficult or impossible to identify through morphology, as is the case for the ecologically important solitary bee fauna.

## Background

The current extinction crisis poses dramatic threats to global diversity [1]. Identification and cataloguing of natural fauna and flora is key to the conservation of biodiversity, but this process is currently hampered by a lack of taxonomic resources [2]. DNA barcoding was suggested as a rapid identification method to catalog the diversity of life [3]. Although initial enthusiasm has been tempered by recognition of its limits in some situations [4], barcoding has tremendous potential to produce more rapid identification of difficult groups and highlight areas of unrecognized diversity [5,6].

The movement to produce large-scale databases of DNA barcodes for animal taxa has resulted in several prominent projects [7,8]. However, attempts to strictly evaluate the accuracy of DNA barcoding, and in particular the 5' cytochrome c oxidase I (COI) fragment typically used [3], have been remarkably limited in the literature. Such evaluation requires testing molecular identification tools (the DNA barcode) against multiple individuals per species, across a species-rich clade, with *a priori *clearly defined species. In contrast, most phylogenetic and barcoding studies utilize either a single individual per species, or select a sample of species from across a larger group, or both. This is especially true for highly diverse groups of invertebrates, where morphological identification is most difficult and recently-diverged species are likely to be present- in short, those in which barcoding is both most likely to be useful, and where it is most likely to encounter difficulties [9]. Most studies looking at these groups have either dealt with attempts to separate cryptic species [10-12]- sometimes with results that conflict depending on the authors' interpretation [13,14]- or have used data sets of relatively well-diverged species that would be expected to be separated by almost any gene [15,16]. When closely-related species have been examined, COI has sometimes failed to discriminate between species that are separable by other means [9,17,18].

Heteroplasmy- the presence of multiple mitochondrial DNA haplotypes in a single organism- has been cited as one of several potential genetic problems for the use of mtDNA for barcoding [19], but no studies have been conducted to examine its practical effects. Compared to nuclear pseudogenes of mtDNA genes (numts), which are more frequently studied [20,21], heteroplasmy is more difficult to control for because multiple haplotypes presumably remain functional and lack any telltale signs in the sequence such as stop codons or frameshift mutations. Heteroplasmy has been extensively studied in humans due to its role in mitochondrial disease [22], and has been documented in insects and other invertebrates. However, aside from genetically unusual groups such as the *Mytilus *bivalves [23] it has generally been poorly studied elsewhere. Although it is often suggested to be more common than thought [14,24], published data are relatively rare. Most evidence of natural heteroplasmy in arthropods has been of length polymorphism in the A-T rich control region [25-28]; in comparison, reported cases of sequence polymorphism in coding genes are sparse [29,28-31]. In honeybees, paternal mtDNA is known to persist at relatively high levels in early stages of development [32], and paternal leakage resulting in heteroplasmy has been documented in *Drosophila *[33] and cicadas [34], indicating a pathway for the origin of heteroplasmy.

This study aims to examine the issues of both heteroplasmy and taxon sampling, by densely sampling a clade of closely-related heteroplasmic species. The Hawaiian *Hylaeus (Nesoprosopis) *bee radiation consists of 60 known species, derived from a single ancestor, with subgeneric relatives in East Asia [35]. They are the only bees native to the Hawaiian Islands, and many are of conservation significance, with 10 species possibly extinct and another 21 threatened to some degree [36]. They are also evolutionarily important, because they apparently include the only kleptoparasitic species of Colletidae [35]. As a group they are among the most widespread Hawaiian insects [37], but most species are fairly specific to one of three habitat zones: wet and mesic forest, coast and dry forest, or dry and subalpine shrubland. All of the taxa have already been well-characterized taxonomically in light of morphological and mtDNA sequence data, and no morphological justification has been found for splitting species with high intraspecific divergence [35]. Previous phylogenetic and biogeographic studies indicate that most or all of the lineages in the group originated on the youngest island of Hawai'i, which is under 1 million years old [38]. Based on this dating, the rate of mtDNA change is extremely high, approximately 15%/million years uncorrected, but the dating is corroborated by a very low degree of differentiation in nuclear genes [39]. In addition, the group is characterized by widespread heteroplasmy in mitochondrial sequences, including a high level of divergence between heteroplasmic haplotypes within individual specimens [38].

Bees in general are a group that would benefit strongly from DNA barcoding. Morphological separation of species is often extremely difficult without previously determined specimens on hand, requiring subjective interpretation of variable characters such as cuticle punctation or coloration [40]. Many species are diagnosed solely or most reliably on the basis of male genitalia or other sex-specific characters, making association of the sexes in similar, sympatric species impossible [35,40,41]. Cryptic species have been recognized based on correlation between DNA sequences and other differences [42,43], although sometimes with only low degrees of genetic divergence [18]. The global decline in pollinators is often cited as a serious threat to both biodiversity and human agriculture [44], and although evidence has mounted that bees have been heavily impacted by human activities, many gaps remain in our knowledge [45]. Partly this is due to a lack of sufficient identification and taxonomic resources, which have been recognized as a major impediment to bee conservation [46] and broader ecological study [41]. Much attention has been focused on honeybees and bumblebees, in part due to their familiarity and cultural importance, but solitary bees include most of the diversity of bees and are more vulnerable to threats such as habitat destruction [47,48]. If DNA barcoding is to play a regular role in identification of adult insects, not just unidentifiable specimens such as juveniles or fragmentary material, then bees are likely to be an important group it is used on.

## Results

### Heteroplasmy

**Figure 1:**
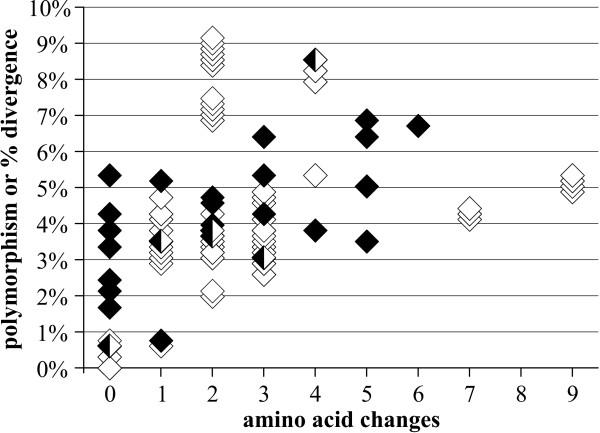
**Polymorphism vs. amino acid changes in heteroplasmic individuals (filled diamonds), compared to intraspecific, interisland pairwise divergence vs. amino acid changes in homoplasmic species (open diamonds)**.

The most striking characteristic of the Hawaiian *Hylaeus *is the high proportion of heteroplasmic species. Twenty-one of the 49 sampled species amplified multiple haplotypes from within individual specimens. In 18 of these species, all individuals tested were heteroplasmic. Of the three remaining species, heteroplasmy was limited to specimens from the Moloka'i population in *H. angustulus*, specimens from the East Maui population in *H. haleakalae*, and 11 out of 13 individuals in *H. unicus*. In some species, e.g. *H. kukui*, *H. setosifrons*, and some populations of *H. connectens*, polymorphic sites in heteroplasmic individuals accounted for 5-6% or more of the total sequence (Fig. 1). However, previous results from cloning [38] and the presence of widely varying chromatogram peak heights suggest that more than two haplotypes are usually present within any one heteroplasmic individual. Consequently, the divergence between any two haplotypes from a heteroplasmic individual is likely to be less than the sum of all variable sites.

**Figure 2:**
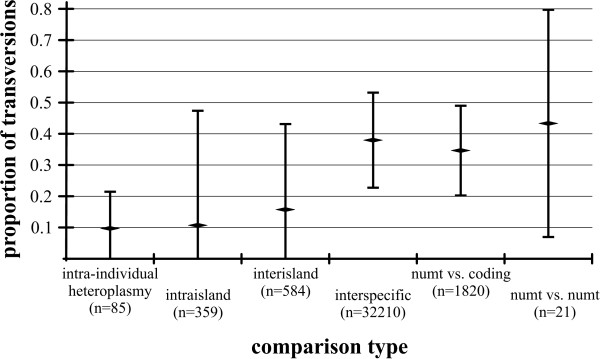
**Average proportion of transversions for sequence comparisons at different biological levels**. For each comparison, data shown are the mean ± 95% confidence intervals.

The pattern of base changes indicates that most cases of polymorphism are due to heteroplasmy rather than nuclear pseudogenes (numts; see below). Although some sequence reads had extremely high levels of polymorphism within heteroplasmic individuals, such changes were overwhelmingly synonymous transitions, similar to intraspecific pairwise comparisons in non-heteroplasmic species (these latter comparisons rely upon *a priori *species definitions, but we note that COI supported these definitions- see below). This can be seen in Fig. 1, where 74% of heteroplasmic sequence polymorphism comparisons (within individuals), and 78% of homoplasmic intraspecific, interisland sequence divergence comparisons resulted in 3 or fewer amino acid changes. Similarly, Fig. 2 shows that the mean proportion of transversions was roughly 0.1 for both intra-individual heteroplasmic sequence comparisons and inter-individual comparisons from within island populations. Interspecific comparisons had a much higher proportion of transversions (Fig. 2), presumably due to the extreme A/T bias (93.6% A/T in third positions) overcoming the greater short-term frequency of transitions. This phenomenon has been frequently observed in insect mtDNA [49]. Comparisons between numts showed a similar pattern to that of interspecific comparisons (Fig. 2). Overall, while there was a trend towards a higher proportion of transversions at a given sequence divergence in numt and interspecific comparisons, in contrast to heteroplasmic sequence comparisons, the amount of variability in the data obscures any potential signal (Figs. 1 and 2). Consequently, the proportion of transversions cannot be used to unambiguously differentiate between heteroplasmic sequences and numts.

The heteroplasmic origin of polymorphism in *Hylaeus *is also supported by results from the mitochondrial enrichment experiments. Chromatograms obtained from mtDNA-enriched extractions of heteroplasmic species were fully identical to those from the controls (total DNA). The enriched samples amplified the single-copy nuclear gene TPI very weakly or not at all, but strongly amplified the multi-copy 28S, while the controls amplified both. This indicates that while some nuclear contamination did occur despite enrichment, it was not sufficient for a single locus to amplify strongly. Only 12 of the ~2,000 numt fragments reported from the honeybee genome are over 640 bp [50]; assuming a similar distribution in *Hylaeus*, numt amplification would be significantly reduced or eliminated by the enrichment. Thus, if polymorphisms were due to the presence of numts, one would expect that at least the relative height of the chromatogram peaks would be altered in enriched samples (as occurred in *H. rugulosus*, discussed below), but this was not the case.

### Numts

Specimens from four species, *H. nivicola*, *H. paradoxicus*, *H. rugulosus*, and *H. specularis*, coamplified sequences that appear to be numts. In *H. paradoxicus *and *H. rugulosus*, the coding sequence and numt were codominant. The coding sequence was cleanly obtained by using the primer combination LCOHym-Pat, suggesting that either only the COI gene was transferred to the nuclear genome, or the tRNA portion of the numt had undergone more extensive change. The other species, *H. nivicola *and *H. specularis*, did not amplify with these primers, but the coding sequence was strongly dominant (the peaks of the *H. specularis *Kauai numt were very weak, and the sequence may not be reliable). Further evidence in *H. rugulosus *came from manipulating PCR conditions, with a PCR annealing temperature of 45°C with LCO-Nancy yielding predominantly the numt, while at 54°C the product was primarily the coding sequence. Finally, the mtDNA-enriched extraction of *H. rugulosus *was still polymorphic, but with the coding sequence rather than the numt predominant, again supporting the conclusion that the alternate sequence is a numt. This enriched sample also amplified 28S but not TPI, confirming that mtDNA enrichment was only partially successful.

**Figure 3:**
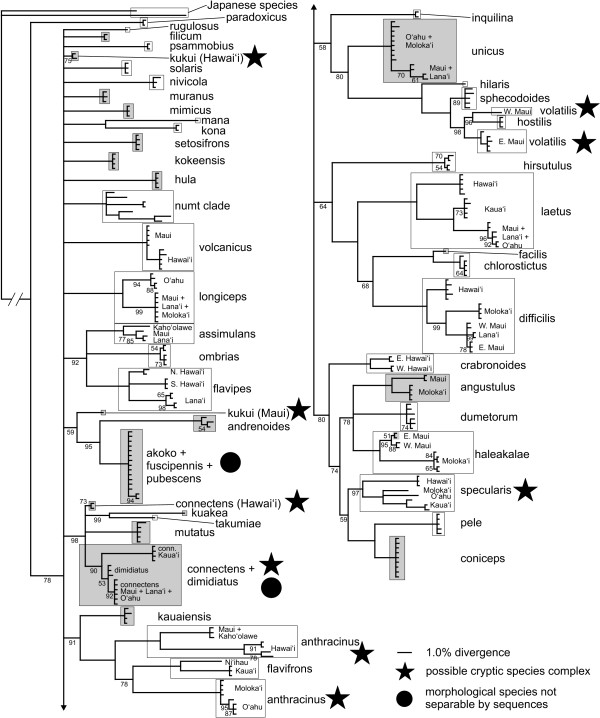
**The Bayesian consensus tree, with posterior probabilities given below branches**. Nodes without values are 100% PP. Shading indicates heteroplasmic species or individuals.

**Figure 4:**
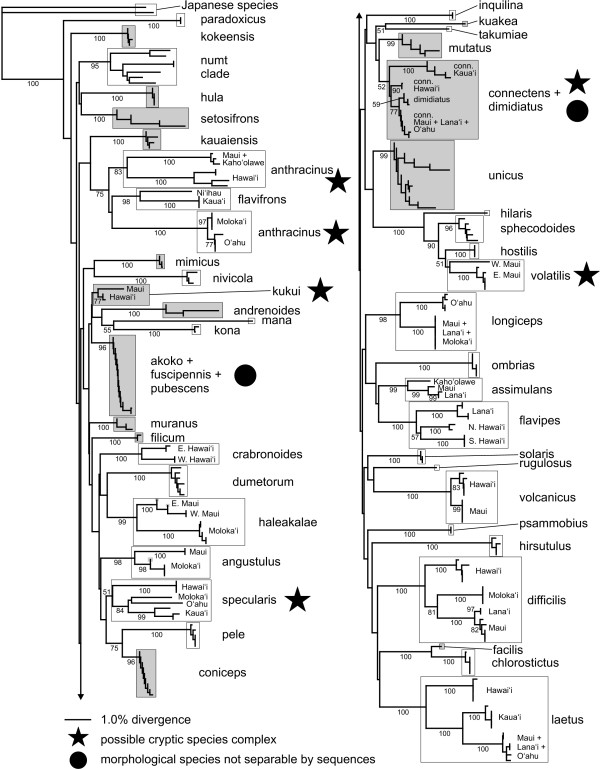
**Neighbor-joining tree with polymorphisms treated as ambiguities**. Bootstrap support values are given below branches (not shown for within-island groupings). Shading indicates heteroplasmic species or individuals.

**Figure 5:**
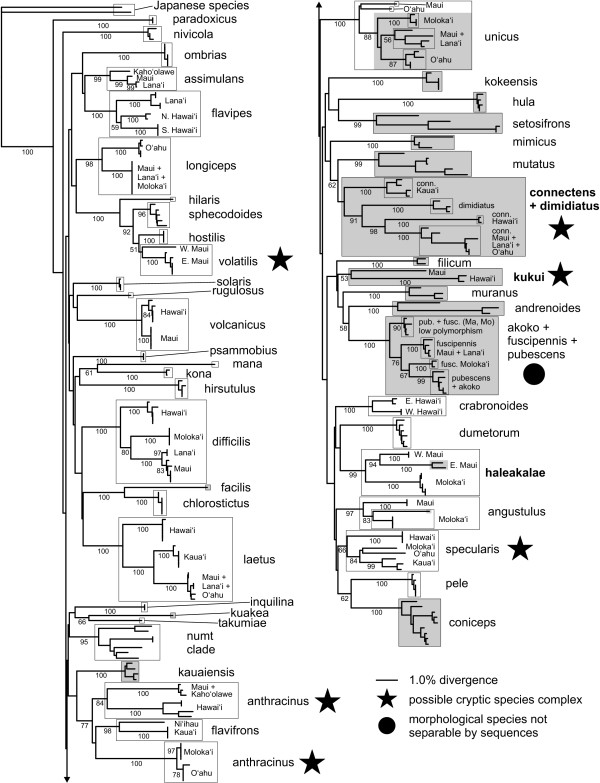
**Neighbor-joining tree with polymorphisms treated as character states**. Bootstrap support values are given below branches (not shown for within-island groupings). Shading indicates heteroplasmic species or individuals.

Remarkably, the numt sequences are highly conserved relative to each other, although with a very high proportion of transversions (Fig. 2) and amino acid changes. This low overall rate of change may be a result of the accelerated base substitution in mtDNA relative to nDNA that is apparently occurring within this radiation [39], similar to the pattern seen in ctenoplectrine bee numts [51]. None of the numts included stop codons, insertions, or deletions, with the possible exception of *H. nivicola*. In both specimens of this species in which the numt amplified, the chromatogram shows signs of a one-base insertion in the reverse sequences only. Finally, the numt sequences form a strongly-supported cluster in all the phylogenetic analyses (Figs. 3, 4 and 5), indicating that the numt probably originated prior to the divergence of at least these four species.

Low-level peaks were also observed to occur among some (but not all) individuals of several other species, e.g., *H. difficilis *and *H. volatilis*. Since these low-level peaks were not strongly associated with third codon positions, they may also indicate weakly-amplifying numts. One of the Japanese species, *H. noomen*, apparently amplifies a numt to the exclusion of the mitochondrial sequence; it is much more divergent from the other numts or coding sequences than any are to each other, differing from *H. globula *in 51 out of 218 amino acid positions (23.4%). Despite this extreme divergence, the *H. noomen *sequence also does not contain any stop codons or indels. Since the presumptive numt of *H. noomen *amplifies cleanly and exclusively, it can be used as an identifying barcode sequence without complication [52]. Due to its extremely high divergence, it is excluded from trees and statistical analyses, but is deposited at GenBank and BOLD.

### Species Separation and Barcoding Identification Accuracy

The aim of this study is to test whether DNA barcoding successfully identifies previously delimited species within a species-rich clade. Consequently, we use the rate at which species are successfully delimited and categorised by COI as compared to their *a priori *definition, which is based primarily on morphology [35], as our measure of success.

There was a lack of higher-level resolution in the Bayesian analysis (Fig. 3), consistent with previous phylogenetic results from other mtDNA gene regions [38] and which is most likely due to rapid saturation of variable bases caused by the high rate of mtDNA evolution (see Introduction, [39]). The NJ analysis, with polymorphisms treated as ambiguities, likewise had short branch lengths (Fig. 4; minimum evolution score 2.16546). This is likely to be due to the coding of polymorphisms as ambiguities. To test this we re-ran the NJ analyses by recoding the polymorphic sites as the expected base sequences in the absence of polymorphism. As expected, the minimum evolution score was larger (2.31436).

Overall, 88% (43/49) of the defined species were recovered as distinct clades on strongly-supported branches. Bayesian posterior probability and neighbor-joining bootstrap were both reliable measures of species delimitation, as nearly all identifiable species had all individuals joined by 100% PP and >95% BS values (the only exception being *H. sphecodoides*, which had 96% BS but only 89% PP). However, heteroplasmy significantly reduced the likelihood of recovering *a priori *defined species- seventy-one percent (15/21) of heteroplasmic, as opposed to 100% (28/28) of homoplasmic species were recovered (Fisher's Exact Test, *p *< 0.01). The species that could not be separated were the *H. fuscipennis-pubescens-akoko *complex (which may be an example of haplotype sharing due to ancestral polymorphism or occasional hybridisation); *H. kukui*, a poorly-known species where the two Hawaii sequences had only weak support as a clade and failed to group with the lone Maui sequence; and the *H. connectens-dimidiatus *complex. All of these species are highly heteroplasmic. In addition, three homoplasmic multi-island species (*H. anthracinus*, *H. specularis*, and *H. volatilis*) each consisted of two or more genetically distinct, highly divergent populations that separately had strong statistical support from both PP and BS, but as a whole were not supported as monophyletic clades. All of these nominal taxa except the *fuscipennis *complex may contain cryptic species that should be further investigated using other methods. At the same time, there was little PP or BS support for multi-species groupings (Figs. 4 and 5), with the exception of the broader *H. volatilis-hostilis-sphecodoides *complex.

The NJ tree with polymorphisms treated as character states (see Methods) had increased branch lengths leading to heteroplasmic species (Fig. 4; minimum evolution score 3.0905), resulting in stronger identification confidence for *H. dimidiatus*, *H. kukui*, and *H. connectens *and *H. haleakalae *populations as determined by BS support values (Fig. 5). This is unsurprising, since treating polymorphism codes as separate character states in effect produces synapomorphies based on polymorphisms shared across a species or island population. The success rate for heteroplasmic species increased to 86% (18/21), which was not significantly different to the 100% recovery rate for homoplasmic species (Fisher's Exact Test, *p *= 0.07). The separation of *H. dimidiatus *is particularly notable, as it is morphologically quite different from *H. connectens *but appears to be derived from within the latter; it has no fixed sequence differences from the Maui Nui + O'ahu *H. connectens*, but possesses a different suite of polymorphic sites. Although *H. fuscipennis*/*pubescens *and *H. unicus *island populations also sorted based on shared polymorphisms, these species included specimens with lower or no polymorphism that cluster together regardless of island origin. Thus, interpreting clusters of sequenced individuals based on polymorphisms as character states should be done with caution and only in comparison to the standard analysis of polymorphisms as ambiguities.

**Figure 6:**
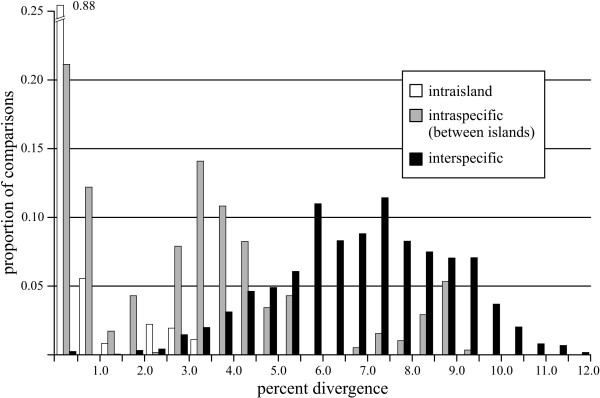
**Histogram of sequence divergence for intraspecific (within-island), intraspecific (between-island), and interspecific pairwise distances (*p*-distance, polymorphisms treated as ambiguities)**. Columns are 0.5% intervals. Percentage divergence between sequences increases from intraisland, interisland (both intraspecific) and interspecific comparisons, but all three show significant overlap.

One of the distinctive features of the Hawaiian *Hylaeus *is the high degree of sequence divergence between island populations of individual species, coupled with generally low variation within islands (Fig. 6). Many island populations were widely divergent within a species, up to over 9%, and 56% of those sampled can be unambiguously determined (Figs. 3, 4 and 5). Hawai'i and Kaua'i, which are relatively distant from their nearest neighbors, always possessed populations with unique haplotypes (with the exception of the heteroplasmic *H. coniceps*), while the islands of Maui Nui (Maui, Moloka'i, Lāna'i, and Kaho'olawe) and O'ahu often shared haplotypes across populations. Island populations predominantly sorted into clusters with under 0.5% internal divergence, but higher intra-island divergence, up to 3.1%, occurred in four species (*H. anthracinus*, *H. crabronoides*, *H. flavipes*, and *H. specularis*). As a result, there is no clear "barcoding gap" [53]- strong overlap exists among intraisland, interisland, and interspecific pairwise distances, although the distribution of the different levels of comparison clearly differ (Fig. 6). Even excluding interspecific comparisons where both sequences are polymorphic due to heteroplasmy (and distance is often highly reduced), all three categories overlap in the region of 2.5-3.1% genetic divergence. The group of high interisland distances in the range of 6.5-9.5% in Fig. 6 consists entirely of comparisons among populations of *H. anthracinus*, a possible species complex. However, when comparisons among potential cryptic species in *H. anthracinus*, *H. connectens*, *H. kukui*, *H. specularis*, and *H. volatilis *are considered as interspecific there is still a large zone of overlap in interspecific and intraspecific (primarily interisland) distance at 2.5-5.5%. Interestingly, the lowest interspecific comparisons occur among the kleptoparasitic species, a pattern seen elsewhere [41].

## Discussion and Conclusions

This is the first study to look at the effectiveness of DNA barcoding in a densely-sampled clade with heteroplasmy. Heteroplasmic species were significantly less likely to be identifiable by standard tree-based DNA barcoding methods, as well as more sophisticated Bayesian methodology, suggesting that heteroplasmy may pose a problem to DNA barcoding approaches to species recognition and delimitation. While all homoplasmic species were successfully recovered in all analyses, only 15 or 18 of the 21 heteroplasmic species were recovered, depending upon the method used. Treating polymorphisms as character states in the NJ analysis was the most effective method for recovering heteroplasmic species, but this analysis is most appropriate with *a priori *defined species, since it will inflate intraspecific branch lengths when polymorphisms are not shared by all individuals of a given species. Consequently, it should be used with caution in studies where DNA barcoding is being used for both species identification and discovery. Only the three species of the *fuscipennis *complex could not be reliably separated using any method. This result suggests that the complex is probably an example of recent divergence with ancestral polymorphism, and the species are likely to be very difficult to differentiate through fixed differences in any mitochondrial or nuclear coding gene. *Hylaeus fuscipennis *and *H. pubescens *are among the few heteroplasmic species that can be found in relative abundance, and may be able to serve as model taxa for future studies of mitochondrial inheritance in this radiation.

In addition, five possible cryptic species complexes are flagged for further investigation, based on weak support for unified clustering of island populations: *H. anthracinus*, *H. connectens*, *H. kukui*, *H. specularis*, and *H. volatilis*. As in previous studies of closely-related species [17,53], the lack of a clear "barcoding gap" in this group (Fig. 6) means that none of these potential complexes can be conclusively determined to consist of multiple species based on COI sequences alone. These results, like many recent studies [6,14,54], reinforce the need for feedback between DNA and traditional taxonomy. DNA sequences are extremely useful to inform taxonomists regarding characters that are not clear- in this case, slight morphological differences between island populations that fall within the typical range of variation for *Hylaeus *species, but that may be diagnostic for "cryptic" species. At the same time, they are often not sufficient in isolation, and additional corroboration by other data (nuclear DNA, morphology, ecology, etc.) is needed.

In addition to the problems in species identification, our analyses also expose other problems that heteroplasmy creates for DNA barcoding as an identification tool. First, unambiguous differentiation between heteroplasmic mtDNA sequences and the numts present here is not possible using direct sequencing. Nor would they be distinguishable through cloning- none of the sequences contained the telltale signs of stop codons, insertions, or deletions (with the possible exception of the presumptive *H. nivicola *numt, noted above). There is a continuum of both sequence and amino acid divergence between definite heteroplasmy (e.g., *H. fuscipennis*) and definite numts (e.g., *H. rugulosus*), and due to the high rate of change in mtDNA relative to nuclear DNA, even unusual amino acid changes are not reliable markers- e.g., the coding sequence of *H. paradoxicus *contains more unique differences than does the numt. The clearest evidence for the nuclear origin of the numts in *Hylaeus *comes from 1) the fact that they are most closely related to each other rather than the coding sequence with which they co- amplify; 2) a higher proportion of nonsynonymous changes (albeit non-significantly so) compared to coding sequences; and 3) the pattern of divergence from each other since transfer to the nuclear genome, particularly a high proportion of non-A/T transversions. However, it would not be possible to distinguish a newly-derived numt that remains close to its coding sequence by using these methods. Indeed, it is entirely possible that some polymorphic species with multiple amino acid changes may be amplifying both heteroplasmic haplotypes and numts.

Second, the fact that a much greater proportion of species were found to be heteroplasmic here than in the previous study- 21 vs. 10 [38]- highlights some of the practical difficulties inherent in barcoding heteroplasmic species. On the biological side, the earlier work primarily used DNA extracted from the abdomen (reproductive organs and/or gut), whereas new extractions prepared for this study used DNA extracted from muscles in the legs. In humans, skeletal muscle is known to consistently accumulate higher levels of some pathogenic mutants than more rapidly-dividing tissues [55]. Although the process is not fully understood, a similar pattern has been found in some *Hylaeus *species, where sequences from muscles were highly polymorphic and those from abdominal tissues much less so, and sometimes nearly clean [56].

Little has been made of how technological issues affect interpretation of DNA sequences, but they are significant when dealing with heteroplasmic individuals. All chromatograms produced by the dye-terminator sequencing method have a certain amount of "background noise," small spurious peaks that do not represent true bases. The earlier sequences were done on an ABI 377 sequencer with a plate gel, which had a relatively high level of background noise. As a result, sequences where all secondary peaks were low were considered to be homoplasmic. When sequencing was done with autoradiographs, assessing unequal polymorphism was even more problematic. In contrast, the newer capillary machines with which the current sequences were produced have a much lower noise level, with the result that heteroplasmy is recognizable even when the non-dominant peak is relatively low. Nevertheless, there is still a great deal of subjectivity in manual editing of chromatograms, and it is here that heteroplasmy poses the greatest difficulty for DNA barcoding. Unlike nuclear DNA, where alleles are present in more or less equal proportions, mtDNA haplotypes may be present in any ratio, and as a result the height of secondary peaks can vary from virtually absent to fully co-dominant. The situation is further complicated by the possibility of large numbers of haplotypes in a single individual, so that polymorphic sites may vary widely among themselves in secondary peak height.

Although these issues in themselves do not appear to be the cause of the species identification problems in Hawaiian *Hylaeus*, they may potentially undermine some of the rationale for broad application of DNA barcoding. First, analysis of heteroplasmic sequences requires much more time both in editing the chromatograms themselves, because a decision must be made on each potentially polymorphic base, and in training the editors, who must be experienced enough to recognize good peaks. Second, even with highly trained editors, it is likely that two people may not call a given sequence exactly identically due to the continuum of secondary peak heights, from codominance through to the level of background noise. Future sequencing technologies may ameliorate some of these problems, or at least allow for simpler quantification of haplotype abundance [57]. Nevertheless, it is important to recognize the limitations inherent in any protocol, and too often this has not been done with regard to DNA barcoding. In a sense, it could be said that barcoding in this situation is more similar to traditional morphological identification- with each specimen evaluated separately, and requiring individual attention from a person with specialized training- than to the original concept of barcoding as the mass processing of thousands of samples by technicians with a relatively low level of expertise [58,59].

Though there is no way to eliminate the difficulties outlined above, they can be mitigated to some degree, particularly when constructing a reference barcode library. Most important is to include multiple sequences from each species or genetically distinct population in order to cover as much genetic diversity as possible, including possible heteroplasmy, within them. It has already been demonstrated that increased within-species sampling reduces species determination error [60]. It is also important to utilize extractions from different tissue types, in order to account for possible haplotype segregation [56,61]. At present, this is rarely done, and the tissue source for barcoding studies is often not specified [62,63]. Performing a tree reconstruction analysis using polymorphisms as character states may help separate species that possess a consistent suite of haplotypes, and therefore a set of shared polymorphisms. We have shown that this method increases identification reliability of highly heteroplasmic species; however, it comes with the caveat that it also exaggerates within-species branch lengths due to differences in detectable polymorphism. As a result, it should always be used together with a standard analysis (with polymorphisms treated as ambiguities).

This work highlights several important areas for future study. First is the need to look broadly at the extent of heteroplasmy. Although relatively infrequently reported, it is known to occur in many groups [24,26,29,30,64]. Furthermore, recent studies suggest that some examples of polymorphism initially presumed to be numts may in fact be heteroplasmy [14,65]. The paucity of published papers on heteroplasmy compared to the massive amount of mtDNA sequence data implies that it is not pervasive in animals, but it may be more common in certain groups than others. It is noteworthy, for example, that three of the groups in which apparently persistent coding sequence heteroplasmy is documented in wild taxa- Hawaiian *Hylaeus*, Indonesian *Chitaura *grasshoppers [29], and Mauritian *Drosophila *[66]- are island endemic species, where adaptive radiation and/or low population sizes may result in increased rates of change [39]. Lice (Phthiraptera), which also have high mutation rates in mtDNA [67] and are known to be be highly heteroplasmic in at least one species [19], are another candidate group for broader study.

Second, our results contrast with those of a recent study that targeted the "barcode sequence" for species discrimination in closely-related bees [18]. In that study [18], COI failed to reliably distinguish between three semi-cryptic species of *Colletes*, which exhibited very low levels of divergence from each other as well as related species, while the nuclear gene EF-1α did contain fixed differences that separated them (albeit still very few). These results suggested that DNA barcoding based on the COI gene might fail in this ecologically important and species rich group. In contrast, our *Hylaeus *data suggest that DNA barcoding based on COI can be highly efficient within the same family of bees (Colletidae), due to dramatically different rates of genetic change. This highlights the difficulty in applying the results of any one study across a broad taxonomic group. These results have important implications for the upcoming Bee-BOL project (see http://www.bee-bol.org), which aims to generate barcodes for all bee species. In looking at these two cases and the future for barcoding in bees, it is important to keep the ultimate goal in perspective. Inevitably, with any single marker, or even a combination of several [68], there will be some species or groups for which it simply does not work, and this has been recognized from the beginning of the DNA barcoding concept [3]. Nevertheless, if a high proportion of bee species can be reliably and relatively rapidly identified by DNA sequences, this would be a substantial improvement over the current situation, where identification relies heavily on variable, subjective, and/or fragile characters such as setation, coloration, cuticle sculpture, and male genitalia [35,40]. While *Hylaeus *and *Colletes *may represent extremes of high and low divergence, additional corroboration from densely-sampled phylogenetic and cryptic-species studies [42,69-71] indicates that the general trend among bees is closer to *Hylaeus*. The success rate in barcoding of a regional continental bee fauna [41] also suggests that relatively few taxa will exhibit low interspecific divergence.

Finally, there is a great deal still to be learned about the evolution of *Hylaeus *in Hawaii. The origin and maintenance of heteroplasmy in the group, and how it relates to its rapid speciation, is an important question for genetics broadly. The fact that heteroplasmy is almost entirely confined to the "wood-nesting clade" [38]- and that it is now known to occur to a greater or lesser degree in 20 of the 27 species examined in that subgroup- suggests a historical basis for the phenomenon. More immediately, the five species identified above as being potentially comprised of multiple cryptic taxa should be further examined. *Hylaeus anthracinus *is of particularly urgent concern, as it is restricted to threatened coastal habitats [36], and the status of the Maui population is unknown. *Hylaeus volatilis *is abundant on East Maui, but the divergent West Maui population is restricted to a single known site; populations on Moloka'i, Lāna'i, and O'ahu, which were likely to be equally distinctive, have not been collected in over 70 years and may have been extirpated. Two others, *H. kukui *and *H. specularis*, appear to be exceptionally rare despite an abundance of their habitat. All exhibit some variation between island populations in characters such as coloration, but not greater than that seen within homosequential populations of other species, and no structural differences have yet been found. Unfortunately, one of the limitations in working with the Hawaiian *Hylaeus *is that many of the heteroplasmic species that it would be most productive to study more intensively (e.g. *H. kukui*, *H. muranus*, *H. setosifrons*) are also extremely rare, and few individuals are available. Perhaps this is not a coincidence, and it is worth investigating whether carrying multiple haplotypes, some of them possibly subtly deleterious [72], may contribute to the competitive disadvantage that *Hylaeus *and other island endemics appear to have relative to alien invasive species. We hope that this study will further the conservation of *Hylaeus *in Hawai'i and bees in general.

## Methods

### Taxon Sampling

Forty-nine of the 60 Hawaiian species of *Hylaeus *were included in the study, along with three Japanese species of subgenus *Nesoprosopis *(Table 1; see Additional File 1 for details). Species were delimited *a priori *using current taxonomy, where morphological differences between species have been shown to match mtDNA sequence divergence [35]. One of the missing species, *H. perkinsianus*, is known to be extant but is endemic to the remote island of Nihoa and no specimens were available; the remaining 10 have not been collected in over 70 years and several are likely to be extinct. In order to account for possible cryptic species, at least one sequence was included from all island populations known to be extant, for a total of 257 individuals from 84 populations. Up to five individuals per island population, from multiple locations if possible, were sequenced to examine intraspecific and intrapopulational variability. However, due to rarity many species and populations are represented by smaller numbers. The term "interisland" will be used to refer to comparisons between sequences of the same species from different island populations.

**Table 1:** Summary of numbers of specimens sequenced for each species and population

		island								
			
species name	author	Ni	Ka	Oa	Mo	La	Kh	Ma	Ha	total
*akoko*	Magnacca & Daly, 2003								**1**	**1**
*andrenoides*	(Perkins, 1899)		**3**							**3**
*angustulus*	(Perkins, 1899)				**4**	×		2		**6**
*anthracinus*	(F. Smith, 1853)			4	4	×	1	2	4	15
*assimulans*	(Perkins, 1899)			×		2	1	1		4
*chlorostictus*	(Perkins, 1899)		5							5
*coniceps*	(Blackburn, 1886)							**4**	**5**	**9**
*connectens*	(Perkins, 1899)		**4**	**3**	×	**1**		**2**	**2**	**12**
*crabronoides*	(Perkins, 1899)								4	4
*difficilis*	(Perkins, 1899)				4	2		5	5	16
*dimidiatus*	(Perkins, 1899)								**3**	**3**
*dumetorum*	(Perkins, 1899)								6	6
*facilis*	(F. Smith, 1879)			×	**1**	×		×		**1**
*filicum*	(Perkins, 1911)								**2**	**2**
*flavifrons*	(Kirby, 1880)	1	3							4
*flavipes*	(F. Smith, 1853)					4		×	5	9
*fuscipennis*	(F. Smith, 1879)			×	**4**	**2**		**3**		**9**
*haleakalae*	(Perkins, 1899)				5			**4**		**9**
*hilaris*	(F. Smith, 1879)				1	×		×		1
*hirsutulus*	(Perkins, 1899)		4							4
*hostilis*	(Perkins, 1899)		3							3
*hula*	(Perkins, 1911)								**4**	**4**
*inquilina*	(Perkins, 1899)								2	2
*kauaiensis*	(Perkins, 1899)		**4**							**4**
*kokeensis*	Magnacca & Daly, 2003		**4**							**4**
*kona*	(Blackburn, 1886)								2	2
*kuakea*	Magnacca & Daly, 2003			**1**						**1**
*kukui*	Magnacca & Daly, 2003							**1**	**2**	**3**
*laetus*	(Perkins, 1899)		5	1	×	2		3	5	16
*longiceps*	(Perkins, 1899)			4	3	2		2		11
*mana*	Magnacca & Daly, 2003			1						1
*mimicus*	Magnacca & Daly, 2003			**3**						**3**
*muranus*	(Warncke, 1970)								**3**	**3**
*mutatus*	(Perkins, 1899)		**5**							**5**
*nivicola*	Meade-Waldo, 1923							3		3
*ombrias*	(Perkins, 1910)								5	5
*paradoxicus*	(Perkins, 1899)								2	2
*pele*	(Perkins, 1911)								5	5
*psammobius*	(Perkins, 1911)							2	×	2
*pubescens*	(Perkins, 1899)								**5**	**5**
*rugulosus*	(Perkins, 1899)								1	1
*setosifrons*	(Perkins, 1899)								**4**	**4**
*solaris*	Magnacca & Daly, 2003		3							3
*specularis*	(Perkins, 1899)		3	1	1				3	8
*sphecodoides*	(Perkins, 1899)								5	5
*takumiae*	Magnacca & Daly, 2003							1		1
*unicus*	(Perkins, 1899)			**5**	**3**	**1**		**4**		**13**
*volatilis*	(F. Smith, 1879)			×	×	×		6		6
*volcanicus*	(Perkins, 1899)							5	5	10

Full details for each specimen, including GenBank accession numbers, are given in Additional File 1. Bold indicates heteroplasmic populations. Island abbreviations: Ka = Kaua'i, Oa = O'ahu, Mo = Moloka'i, La = Lāna'i, Ma = Maui, Ha = Hawai'i. Historic populations not included in this study are denoted by an "×".

### DNA Extraction

Total DNA was extracted using the phenol-chloroform-isoamyl alcohol method [73] as described by Magnacca and Danforth [38], or using the DNeasy Blood & Tissue extraction kit (Qiagen Inc.), following the manufacturer's protocol. In general, DNA taken from specimens extracted using the former method came from the whole body, thoracic musculature, or reproductive organs, while those extracted with the Qiagen kit used the mid and hind right legs (see Additional File 1 for details). In order to account for the possibility of nonrandom haplotype segregation [56], at least one extraction each from muscles and abdominal tissue (primarily reproductive organs with some gut) was used for all species represented by more than one individual. Both muscle and abdominal tissue was extracted by both methods for some species, and no effect of the extraction method was seen in sequencing results even in heteroplasmic species exhibiting segregation.

Mitochondrial enrichment, using the method of Saito et al. [74], was performed on specimens of four heteroplasmic species, *H. coniceps*, *H. connectens*, *H. fuscipennis*, and *H. pubescens*, and one containing a presumptive numt, *H. rugulosus*. Thoracic muscle (~1-2 mg) was homogenized with a plastic pestle in 1 ml of buffer containing 0.25 m sucrose, 10 mm EDTA, and 30 mm Tris (pH 7.6) and centrifuged at 1000 g for 5 min at 4°. The supernatant was retained and centrifugation repeated 1-3 times. The final supernatant was centrifuged at 12,000 g for 10 min at 4° to pellet mitochondria, the supernatant discarded, and the pellet extracted using the Qiagen kit as above. DNA was also extracted from the pellet from the first centrifugation, containing the nuclei and unbroken cells, as a control. Since this fraction still contains large quantities of mtDNA in the unbroken cells, it is not expected to be nuclear-enriched relative to standard extractions.

### PCR and Sequencing

Primer sequences are listed in Table 2. The "standard" barcoding fragment of cytochrome oxidase I was amplified using a version of the commonly-used primer LCO [75], modified for use in Hymenoptera, with a shortened version of "Nancy" [76] as the reverse (due to an ordering error, the last 3 bases of the latter were accidentally left off, but this version worked better than the longer sequence). These would be called C1-J-1514 and C1-N-2194 under the Simon et al. [76] naming scheme. Nancy partially overlaps with primer HCO, often used in combination with LCO, but also gives a slight overlap with sequences generated with the primer pair Jerry-Pat [38]. The combination of LCOHym-Nancy also gave slightly better yields than LCOHym-HCO in *Hylaeus*. PCR was run using standard Taq (Invitrogen Corp.) with the following program: a starting denaturation at 94° for 180 seconds, followed by 35 cycles of 94° for 30 s, 48° for 45 s, and 72° for 60 s, concluding with a final extension at 72° for 240 s. One species, *H. mana*, proved to be intractable with this primer combination and was amplified using a novel primer, C1-J-1580, as the forward primer. TL2-N-3014 "Pat" [76] was used as the reverse for two species that amplified numts, with an annealing temperature of 46° (*H. paradoxicus*) or 41° (*H. rugulosus*) and an extension time of 105 s. MtDNA-enriched extractions were tested for the presence of nuclear DNA by attempting to amplify 28S rDNA and the single-copy nuclear gene triosephosphate isomerase (TPI).

**Table 2:** Primers used in this study.

locus	primer	source	sequence
COI	LCO_Hym (C1-J-1514)	[75]	5'-TATCAACCAATCATAAAGATATTGG-3'
	C1-J-1580	new	5'-ACATCTTTAAGAATAATTATTCG-3'
	Nancy-short (C1-N-2194)	[76]	5'-CCCGGTAAAATTAAAATATAAAC-3'
	Pat (TL2-N-3014)	[76]	5'-TCCAATGCACTAATCTGCCATATTA-3'
			
28S	28S for (D2-3665F)	[82]	5'-AAGAGAGAGTTCAAGAGTACGTG-3'
	28Srev (D3-4283R)	[82]	5'-TAGTTCACCATCTTTCGGGTCCC-3'
			
TPI	L1F	[83]	5'-CTKCGTBGGNGGNAACTGGAAGATGA-3'
	L1R	[83]	5'-CCRATRGCCCANACNGGYTCRTA-3'

PCR products were sent for sequencing by Macrogen (Seoul, South Korea) or sequenced in the School of Natural Sciences, Trinity College; both utilized ABI 3130xl capillary automated sequencers (Applied Biosystems Inc.). The PCR primers were used for sequencing. Most were sequenced from both directions, although sequences were of high enough quality that only one direction was necessary. All polymorphic species were sequenced from both directions in order to ensure correct basecalling. Since the point of this study is to evaluate the use of "barcoding" in the sense of rapid direct sequencing for identification, no attempt was made to obtain clean haplotype sequences of heteroplasmic individuals via cloning (see Discussion). Chromatograms were edited using FinchTV (Geospiza Inc.). Sequences were submitted to GenBank (accession nos. FJ411517-FJ11791), and sequences, trace files, and specimen data submitted to BOLD (http://www.barcodinglife.org, project "*Hylaeus *of Hawaii"). For species where nuclear pseudogenes (numts) were known or inferred to be co-amplified, three sequences were submitted: the original polymorphic read, the coding sequence, and the sequenced (*H. rugulosus*) or inferred (other species) numt sequence (due to GenBank submission requirements, inferred numt sequences are available only at BOLD).

### Sequence Analysis

Alignment of sequences was trivial as no gaps were present. After trimming the ends, a sequence of 654 base pairs was used for analysis (598 for *H. mana*). For tree construction, a 2-partition Bayesian analysis was performed with MrBayes 3.1.2 [77]. Using three partitions (one for each codon position) produced a slightly lower overall -lnL but resulted in failure of parameters to converge due to the low number of changes in second positions. Models for each partition were selected using MrModeltest 2.2 [78]; these were determined to be GTR+I+G for codon positions 1 + 2, and GTR+G for codon position 3. The analysis was run for 4 million generations with a burnin of 1 million. For comparison with previous barcoding studies, trees were also generated using the neighbor-joining pairwise distance algorithm in PAUP* 4.0b10 [79], using uncorrected ("*p*") distances, and neighbor-joining bootstrap performed with 10,000 replicates. The Kimura 2-parameter model is frequently used without justification [11,13,63]; due to the strong A/T bias of the sequences here it is inappropriate, since A-T transversions occur at a far higher rate than others. In order to take widespread heteroplasmy into account, the matrix was analyzed two ways: under the standard method of treating polymorphic sites as ambiguities, and treating polymorphism codes (R, Y, etc.) as separate character states. The latter allows consistent polymorphisms to increase branch lengths and therefore make separation of species and populations easier. It also increases apparent intraspecific variability due to the inherent subjectivity in determining heteroplasmic polymorphism (see Discussion). Because PAUP interprets data in this format as non-DNA, the polymorphisms as character states analysis was performed using the mean number of pairwise differences model. Although several character-based, non-distance methods have been developed [52,80], and are more philosophically sound in species delimitation [81], they have not been shown to have significant advantages in identification accuracy where alignment is not an issue. Tests also indicated difficulties with handling of polymorphisms in character-based programs. We note that this second test, with polymorphism codes being treated as character states, is more appropriate with *a priori *defined species.

To measure the success rate of DNA barcoding, we used the rate at which the defined species were successfully delimited into statistically supported monophyletic groupings. Clades were considered to have high support if they had a Bayesian posterior probability value of over 90 and a bootstrap value of over 75. We compared the success in recovering homoplasmic and heteroplasmic species using a Fisher's Exact Test.

## Authors' contributions

KM collected specimens and performed genetic sequencing and analysis, and wrote the manuscript. MJFB conceived of the study and assisted in drafting the manuscript. Both authors designed the study and have read and approved the manuscript.

## Supplementary Material

Additional file 1 Collection details and GenBank accession numbers for all specimens included in this study. Extraction codes beginning with C were done with the phenol-chloroform-isoamyl alcohol method, those beginning with T were performed with the Qiagen kit (see Methods); "n" and "p" appended to the extraction code denote numt and composite numt/coding sequences respectively. FR = Forest Reserve; HALE = Haleakalā National Park; HAVO = Hawai'i Volcanoes National Park; KFU = Kona Forest Unit of Hakalau National Wildlife Refuge; NAR = Natural Area Reserve; NHP = National Historical Park; SP = State Park; WS = Wildlife Sanctuary. Click here for file

## References

[B1] Baillie JEM Hilton-Taylor C Stuart SN (Eds) IUCN Red List of Threatened Species: A Global Species Assessment 2004 Gland Switzerland and Cambridge UK: IUCN

[B2] Mace GM The role of taxonomy in species conservation Phil Trans R Soc B 2004 359 711 719 10.1098/rstb.2003.1454 15253356 PMC1693347

[B3] Hebert PDN Cywinska A Ball SL DeWaard JR Biological identifications through DNA barcodes Proc R Soc B-BiolSci 2003 270 313 321 10.1098/rspb.2002.2218 PMC1691236 12614582

[B4] Monaghan MT Balke M Pons J Vogler AP Beyond barcodes: complex DNA taxonomy of a South Pacific island radiation Proc R Soc B-BiolSci 2006 273 887 893 10.1098/rspb.2005.3391 PMC1560222 16618684

[B5] Barber P Boyce SL Estimating diversity of Indo-Pacific coral reef stomatopods through DNA barcoding of stomatopod larvae Proc R Soc B-BiolSci 2006 273 2053 2061 10.1098/rspb.2006.3540 PMC1635474 16846913

[B6] Smith MA Rodriguez JJ Whitfield JB Deans AR Janzen DH Hallwachs W Hebert PDN Extreme diversity of tropical parasitoid wasps exposed by iterative integration of natural history DNA barcoding morphology, and collections Proc Nat Acad Sci USA 2008 105 12359 12364 10.1073/pnas.0805319105 18716001 PMC2518452

[B7] Kerr KCR Stoeckle MY Dove CJ Weigt LA Francis CM Hebert PDN Comprehensive DNA barcode coverage of North American birds Mol Ecol Notes 2007 7 535 543 10.1111/j.1471-8286.2007.01670.x 18784793 PMC2259444

[B8] Costa FO Carvalho GR The Barcode of Life Initiative: synopsis and prospective societal impacts of DNA barcoding of fish Genom Soc Pol 2007 3 29 40

[B9] Skevington JH Kehlmaier C Stahls G DNA Barcoding: Mixed results for big-headed flies (Diptera: Pipunculidae) Zootaxa 2007 1 26

[B10] Witt JDS Threloff DL Hebert PDN DNA barcoding reveals extraordinary cryptic diversity in an amphipod genus: implications for desert spring conservation Mol Ecol 2006 15 3073 3082 16911222 10.1111/j.1365-294X.2006.02999.x

[B11] Smith MA Woodley NE Janzen DH Hallwachs W Hebert PDN DNA barcodes reveal cryptic host-specificity within the presumed polyphagous members of a genus of parasitoid flies (Diptera : Tachinidae) Proc Nat Acad Sci USA 2006 103 3657 3662 10.1073/pnas.0511318103 16505365 PMC1383497

[B12] Smith MA Wood DM Janzen DH Hallwachs W Hebert PDN DNA barcodes affirm that 16 species of apparently generalist tropical parasitoid flies (Diptera, Tachinidae) are not all generalists Proc Nat Acad Sci USA 2007 104 4967 4972 10.1073/pnas.0700050104 17360352 PMC1821123

[B13] Hebert PDN Penton EH Burns JM Janzen DH Hallwachs W Ten species in one: DNA barcoding reveals cryptic species in the neotropical skipper butterfly Astraptes fulgerator Proc Nat Acad Sci USA 2004 101 14812 14817 10.1073/pnas.0406166101 15465915 PMC522015

[B14] Brower AVZ Problems with DNA barcodes for species delimitation: 'ten species' of Astraptes fulgerator reassessed (Lepidoptera : Hesperiidae) Syst Biodivers 2006 4 127 132 10.1017/S147720000500191X

[B15] Costa FO deWaard JR Boutillier J Ratnasingham S Dooh RT Hajibabaei M Hebert PDN Biological identifications through DNA barcodes: the case of the Crustacea Can J Fish Aquat Sci 2007 64 272 295 10.1139/F07-008

[B16] Greenstone MH Rowley DL Heimbach U Lundgren JG Pfannenstiel RS Rehner SA Barcoding generalist predators by polymerase chain reaction: carabids and spiders Mol Ecol 2005 14 3247 3266 10.1111/j.1365-294X.2005.02628.x 16101789

[B17] Kaila L Stahls G DNA barcodes: Evaluating the potential of COI to differentiate closely related species of Elachista (Lepidoptera : Gelechioidea : Elachistidae) from Australia Zootaxa 2006 1170 1 26

[B18] Kuhlmann M Else GR Dawson A Quicke DLJ Molecular, biogeographical and phenological evidence for the existence of three western European sibling species in the *Colletes succinctus *group (Hymenoptera: Apidae) Org Divers Evol 2007 7 155 165 10.1016/j.ode.2006.04.001

[B19] Rubinoff D Cameron S Will K A genomic perspective on the shortcomings of mitochondrial DNA for "barcoding" identification J Heredity 2006 97 581 594 10.1093/jhered/esl036 17135463

[B20] Bensasson D Zhang D-X Hewitt GM Frequent assimilation of mitochondrial DNA by grasshopper nuclear genomes Mol Biol Evol 2000 17 406 415 10723741 10.1093/oxfordjournals.molbev.a026320

[B21] Song H Buhay JE Whiting MF Crandall KA Many species in one: DNA barcoding overestimates the number of species when nuclear mitochondrial pseudogenes are coamplified Proc Nat Acad Sci USA 2008 105 13486 13491 10.1073/pnas.0803076105 18757756 PMC2527351

[B22] Chinnery PF Turnbull DM Mitochondrial DNA mutations in the pathogenesis of human disease Mol Med Today 2000 6 425 432 10.1016/S1357-4310(00)01805-0 11074368

[B23] Skibinski DOF Gallagher C Benyon CM Mitochondrial DNA inheritance Nature 1994 368 817 818 8159240 10.1038/368817b0

[B24] Kmiec B Woloszynksa M Janska H Heteroplasmy as a common state of mitochondrial genetic information in plants and animals Curr Genet 2006 50 149 159 10.1007/s00294-006-0082-1 16763846

[B25] Solignac M Monnerot M Mounolou J-C Mitochondrial DNA heteroplasmy in *Drosophila mauritiana* Proc Nat Acad Sci USA 1983 80 6942 6946 10.1073/pnas.80.22.6942 6316335 PMC390102

[B26] Nardi F Caparelli A Fanciulli PP Dallai R Frati F The complete mitochondrial DNA sequence of the basal hexapod *Tetrodontophora bielanensis*: evidence for heteroplasmy and tRNA translocations Mol Biol Evol 2001 18 1293 1304 11420368 10.1093/oxfordjournals.molbev.a003914

[B27] Boyce TM Zwick ME Aquadro CF Mitochondrial DNA in the bark weevils:size structure and heteroplasmy Genetics 1989 123 825 836 2612897 10.1093/genetics/123.4.825 PMC1203892

[B28] Kann LM Rosenblum EB Rand DM Aging, mating and the evolution of mtDNA heteroplasmy in *Drosophila melanogaster* Proc Nat Acad Sci USA 1998 95 2372 2377 10.1073/pnas.95.5.2372 9482892 PMC19350

[B29] Walton C Butlin RK A phylogeny for grasshoppers of the genus *Chitaura *(Orthoptera: Acrididae) from Sulawesi Indonesia, based on mitochondrial DNA sequence data Biol J Linn Soc 1997 62 365 382

[B30] Frey JE Frey B Origin of intra-individual variation in PCR-amplified mitochondrial cytochrome oxidase I of *Thrips tabaci *(Thysanoptera: Thripidae): mitochondrial heteroplasmy or nuclear integration? Hereditas 2004 140 92 98 10.1111/j.1601-5223.2004.01748.x 15061785

[B31] Paduan KdS Ribolla PEM Mitochondrial DNA polymorphism and heteroplasmy in populations of *Aedes aegypti *in Brazil J Med Entomol 2008 45 59 67 10.1603/0022-2585(2008)45[59:MDPAHI]2.0.CO;2 18283943

[B32] Meusel MS Moritz RFA Transfer of paternal mitochondrial DNA during fertilization of honeybee (*Apis mellifera *L.) eggs Curr Genet 1993 24 539 543 10.1007/BF00351719 8299176

[B33] Kondo R Satta Y Matsuura ET Ishiwa H Takahata N Chigusa SI Incomplete maternal transmission of mitochondrial DNA in *Drosophila* Genetics 1990 126 657 663 2249764 10.1093/genetics/126.3.657 PMC1204221

[B34] Fontaine KM Cooley JR Simon C Evidence for paternal leakage in hybrid periodical cicadas (Hemiptera: Magicicada spp.) PLoS ONE 2007 2 e892 10.1371/journal.pone.0000892 17849021 PMC1963320

[B35] Daly HV Magnacca KN Insects of Hawaii. Hawaiian Hylaeus (Nesoprosopis) Bees (Hymenoptera: Apoidea) 2003 17 Honolulu: University of Hawaii Press

[B36] Magnacca KN Conservation status of the native bees of Hawaii *Hylaeus (Nesoprosopis) *(Hymenoptera: Apoidea) Pac Sci 2007 61 173 190 10.2984/1534-6188(2007)61[173:CSOTEB]2.0.CO;2

[B37] Perkins RCL Sharp D Introduction Fauna Hawaiiensis 1913 1 London: Cambridge University Press i ccxxvii

[B38] Magnacca KN Danforth BN Evolution and biogeography of native Hawaiian *Hylaeus *bees (Hymenoptera: Colletidae) Cladistics 2006 22 393 411 10.1111/j.1096-0031.2006.00119.x

[B39] Magnacca KN Danforth BN Low nuclear DNA variation supports a recent origin of Hawaiian *Hylaeus *bees (Hymenoptera: Colletidae) Mol Phylogenet Evol 2007 43 908 915 10.1016/j.ympev.2006.09.004 17049277

[B40] Mitchell TB Bees of the eastern United States vol. 1 Agr Expt Sta Tech Bull 141 Raleigh NC: North Carolina 1960

[B41] Sheffield CS Hebert PDN Kevan PG Packer L DNA barcoding a regional bee (Hymenoptera: Apoidea) fauna and its potential for ecological studies Molecular Ecology Resources 2009 9 196 207 10.1111/j.1755-0998.2009.02645.x 21564979

[B42] Murray TE Fitzpatrick U Brown MJF Paxton RJ Cryptic species diversity in a widespread bumble bee complex revealed using mitochondrial DNA RFLPs Conserv Genet 2008 9 653 666 10.1007/s10592-007-9394-z

[B43] Bertsch A Schweer H Titze A Tanaka H Male labial gland secretions and mitochondrial DNA markers support species status of *Bombus cryptarum *and *B. magnus *(Hymenoptera, Apidae) Insect Soc 2005 52 45 54 10.1007/s00040-004-0761-1

[B44] Cane JH Tepedino VJ Causes and extent of declines among native North American invertebrate pollinators: detection evidence, and consequences Cons Ecol 2001 5 1

[B45] Brown MJF Paxton RJ The conservation of bees: a global perspective Apidologie 2009 40 410 416 10.1051/apido/2009019

[B46] FAO A Contribution to the International Initiative for the Conservation and Sustainable Use of Pollinators: Rapid Assessment Of Pollinators' Status 2008 Rome: United Nations Food and Agriculture Organization

[B47] Kremen C Williams NM Thorp RW Crop pollination from native bees at risk from agricultural intensification Proc Nat Acad Sci USA 2002 99 16812 16816 10.1073/pnas.262413599 12486221 PMC139226

[B48] Steffan-Dewenter I Potts SG Packer L Pollinator diversity and crop pollination services are at risk Trends Ecol Evol 2005 20 651 652 10.1016/j.tree.2005.09.004 16701452

[B49] Brown JM Pellmyr O Thompson JN Harrison RG Phylogeny of *Greya *(Lepidoptera: Prodoxidae), based on nucleotide sequence variation in mitochondrial cytochrome oxidase I and II: congruence with morphological dat Mol Biol Evol 1994 11 128 141 8121281 10.1093/oxfordjournals.molbev.a040087

[B50] Pamilo P Viljakainen L Viljakainen A Exceptionally high density of NUMTs in the honeybee genome Mol Biol Evol 2007 24 1340 1346 10.1093/molbev/msm055 17383971

[B51] Schaefer H Renner SS A phylogeny of the oil bee tribe Ctenoplectrini (Hymenoptera: Anthophila) based on mitochondrial and nuclear data: Evidence for Early Eocene divergence and repeated out-of-Africa dispersal Mol Phylogenet Evol 2008 47 799 811 10.1016/j.ympev.2008.01.030 18353689

[B52] Little DP Stevenson DW A comparison of algorithms for the identification of specimens using DNA barcodes: examples from gymnosperms Cladistics 2007 23 1 21 10.1111/j.1096-0031.2006.00126.x 34905841

[B53] Meyer CP Paulay G DNA barcoding: Error rates based on comprehensive sampling PLoS Biol 2005 3 2229 2238 10.1371/journal.pbio.0030422 PMC1287506 16336051

[B54] DeSalle R Egan MG Siddall M The unholy trinity: taxonomy species delimitation and DNA barcoding Phil Trans R Soc B 2005 360 1905 1916 10.1098/rstb.2005.1722 16214748 PMC1609226

[B55] Chinnery PF Modulating heteroplasmy Trends Gen 2002 18 173 176 10.1016/S0168-9525(01)02636-1 11932010

[B56] Magnacca KN Brown MJF Tissue segregation of mitochondrial haplotypes in heteroplasmic Hawaiian bees: implications for DNA barcoding Molecular Ecology Resources 2009 10 60 68 10.1111/j.1755-0998.2009.02724.x 21564991

[B57] White HE Durston VJ Seller A Fratter C Harvey JF Cross NCP Accurate detection and quantitation of heteroplasmic mitochondrial point mutations by pyrosequencing Genet Test 2005 9 190 199 10.1089/gte.2005.9.190 16225398

[B58] Hebert PDN Gregory TR The promise of DNA barcoding for taxonomy Syst Biol 2005 54 852 859 10.1080/10635150500354886 16243770

[B59] Hajibabaei M DeWaard JR Ivanova NV Ratnasingham S Dooh RT Kirk SL Mackie PM Hebert PDN Critical factors for assembling a high volume of DNA barcodes Phil Trans R Soc B 2005 360 1959 1967 10.1098/rstb.2005.1727 16214753 PMC1609220

[B60] Matz MV Nielsen R A likelihood ratio test for species membership based on DNA sequence data Phil Trans R Soc B 2005 360 1969 1974 10.1098/rstb.2005.1728 16214754 PMC1609224

[B61] Jenuth JP Peterson AC Shoubridge EA Tissue-specific selection for different mtDNA genotypes in heteroplasmic mice Nat Genet 1997 16 93 95 10.1038/ng0597-93 9140402

[B62] Smith MA Fisher BL Hebert PDN DNA barcoding for effective biodiversity assessment of a hyperdiverse arthropod group: the ants of Madagascar Phil Trans R Soc B 2005 360 1825 1834 10.1098/rstb.2005.1714 16214741 PMC1609228

[B63] Hajibabaei M Janzen DH Burns JM Hallwachs W Hebert PDN DNA barcodes distinguish species of tropical Lepidoptera Proc Nat Acad Sci USA 2006 103 968 971 10.1073/pnas.0510466103 16418261 PMC1327734

[B64] Shigenobu Y Saitoh K Hayashizaki K Ida H Nonsynonymous site heteroplasmy in fish mitochondrial DNA Genes Genet Syst 2005 80 297 301 10.1266/ggs.80.297 16284423

[B65] Sword GA Senior LB Gaskin JF Joern A Double trouble for grasshopper molecular systematics: intra-individual heterogeneity of both mitochondrial 12S-valine-16S and nuclear internal transcribed spacer ribosomal DNA sequences in *Hesperotettix viridis *(Orthoptera : Acrididae) Syst Entomol 2007 32 420 428 10.1111/j.1365-3113.2007.00385.x

[B66] Satta Y Toyohara N Ohtaka C Tatsuno Y Watanabe TK Matsuura ET Chigusa SI Takahata N Dubious maternal inheritance of mitochondrial DNA in *D. simulans *and evolution of *D. mauritiana* Genet Res Camb 1988 52 1 6 10.1017/S0016672300027245

[B67] Johnson KP Cruickshank RH Adams RJ Smith VS Page RDM Clayton DH Dramatically elevated rate of mitochondrial substitution in lice (Insecta: Phthirapera) Mol Phylogenet Evol 2003 26 231 242 10.1016/S1055-7903(02)00342-1 12565034

[B68] Kress WJ Wurdack KJ Zimmer EA Weigt LA Janzen DH Use of DNA barcodes to identify flowering plants Proc Nat Acad Sci USA 2005 102 8369 8374 10.1073/pnas.0503123102 15928076 PMC1142120

[B69] Danforth BN Mitchell PL Packer L Mitochondrial DNA differentiation between two cryptic *Halictus *(Hymenoptera: Halictidae) species Ann Entomol Soc Am 1998 91 387 391

[B70] Danforth BN Phylogeny of the bee genus *Lasioglossum *(Hymenoptera: Halictidae) based on mitochondrial COI sequence data Syst Entomol 1999 24 377 393 10.1046/j.1365-3113.1999.00087.x

[B71] Gibbs J Integrative taxonomy identifies new (and old) species in the *Lasioglossum (Dialictus) tegulare *(Robertson) species group (Hymenoptera, Halictidae) Zootaxa 2009 2032 1 38

[B72] Liau WS Gonzalez-Serricchio AS Deshommes C Chin K LaMunyon CW A persistent mitochondrial deletion reduces fitness and sperm performance in heteroplasmic populations of *C. elegans* BMC Genetics 2007 8 8 10.1186/1471-2156-8-8 17394659 PMC1852114

[B73] Doyle JJ Doyle JL Isolation of plant DNA from fresh tissue Focus 1990 12 13 15

[B74] Saito S Tamura K Aotsuka T Replication origin of mitochondrial DNA in insects Genetics 2005 171 1695 1705 10.1534/genetics.105.046243 16118189 PMC1456096

[B75] Folmer O Hoeh W Lutz R Vrijenhoek R DNA primers for amplification of mitochondrial cytochrome C oxidase subunit I from diverse metazoan invertebrates Mol Marine Biol Biotech 1994 3 294 299 7881515

[B76] Simon C Frati F Beckenbach A Crespi B Liu H Flook P Evolution, weighting and phylogenetic utility of mitochondrial gene sequences and a compilation of conserved polymerase chain reaction primers Ann Entomol Soc Am 1994 87 651 701

[B77] Huelsenbeck JP Ronquist F MRBAYES: Bayesian inference of phylogenetic trees Bioinformatics 2001 17 754 755 10.1093/bioinformatics/17.8.754 11524383

[B78] Nylander JAA MrModeltest v2.2. Program distributed by the author. Evolutionary Biology Centre Uppsala University 2004

[B79] Swofford DL PAUP*: Phylogenetic Analysis Using Parsimony (*and other methods), version 4.0b10. Sinauer Associates Sunderland, Massachusetts 200318428704

[B80] Rach J DeSalle R Sarkar IN Schierwater B Hadrys H Character-based DNA barcoding allows discrimination of genera species and populations in Odonata Proc R Soc B-BiolSci 2008 275 237 247 10.1098/rspb.2007.1290 PMC2212734 17999953

[B81] Kelly RP Sarkar IN Eernisse DJ Desalle R DNA barcoding using chitons (genus Mopalia) Mol Ecol Notes 2007 7 177 183 10.1111/j.1471-8286.2006.01641.x

[B82] Mardulyn P Whitfield JB Phylogenetic signal in the COI 16S, and 28S genes for inferring relationships among genera of microgastrinae (Hymenoptera: Braconidae): Evidence of a high diversification rate in this group of parasitoids Mol Phylogenet Evol 1999 12 282 294 10.1006/mpev.1999.0618 10413623

[B83] Fischer WM The fungal origins of Microsporidia: phylogenies from 70 kd heat-shock protein and small-subunit ribosomal RNA Ph.D 2001 Indiana University Department of Biology

