# Supplementary material for: Mitochondrial heteroplasmy and DNA barcoding in Hawaiian Hylaeus (Nesoprosopis) bees (Hymenoptera: Colletidae)
Source: BMC Evol Biol. 2010 Jun 11;10:174. doi: 10.1186/1471-2148-10-174 (PMC2891727; doi:10.1186/1471-2148-10-174)
Supplement: Additional file 1 — Collection details and GenBank accession numbers for all specimens included in this study. Extraction codes beginning with C were done with the phenol-chloroform-isoamyl alcohol method, those beginning with T were performed with the Qiagen kit (see Methods); "n" and "p" appended to the extraction code denote numt and composite numt/coding sequences respectively. FR = Forest Reserve; HALE = Haleakalā National Park; HAVO = Hawai'i Volcanoes National Park; KFU = Kona Forest Unit of Hakalau National Wildlife Refuge; NAR = Natural Area Reserve; NHP = National Historical Park; SP = State Park; WS = Wildlife Sanctuary. [file 1471-2148-10-174-S1.DOC]

Additional File 1. Collection details and GenBank accession numbers for all specimens included in this study.

|  | BOLD |  |  |  |  | Region/ |  |  |  |  |  |
| --- | --- | --- | --- | --- | --- | --- | --- | --- | --- | --- | --- |
| Species | Process ID | GenBank | Extr. | Sex | Tissue | Island | Locality | Latitude | Longitude | Date | Collector |
| *Hylaeus globula* | HYLHI002-08 | FJ411626 | C208 | M | whole body | Masuda-shi | Inohara-kogen, Yokota-cho | 34.6 | 131.8 | 10 Oct 1999 | Y. Maeta |
| *Hylaeus insularum* | HYLHI003-08 | FJ411650 | C209 | M | whole body | Amami Oshima | Kuji, Setouchi-cho | 28.2 | 129.2 | 27 Mar 1999 | Y. Maeta |
| *Hylaeus noomen* | HYLHI276-08 | FJ411711 | C211 | M | body | Izumo-shi | Sotozono | 35.35 | 132.67 | 30 May 1997 | Y. Maeta |
| *Hylaeus akoko* | HYLHI004-08 | FJ411517 | C245 | M | genitalia | Hawai‘i | Pu‘u Wa‘awa‘a WS | 19.7362 | -155.8402 | 1 Aug 2002 | K. Magnacca |
| *Hylaeus andrenoides* | HYLHI005-08 | FJ411520 | C79 | M | genitalia | Kaua‘i | Alaka‘i Swamp Trail | 22.1362 | -159.6256 | 21 Aug 1999 | K. Magnacca |
|  | HYLHI006-08 | FJ411519 | T57 | M | 2 legs | Kaua‘i | Pa‘aiki Valley | 22.1293 | -159.6924 | 2 Jul 2000 | K. Magnacca |
|  | HYLHI007-08 | FJ411518 | T58 | F | 2 legs | Kaua‘i | Mōhihi Trail | 22.1133 | -159.5872 | 8 Jul 2000 | K. Magnacca |
| *Hylaeus angustulus* | HYLHI008-08 | FJ411526 | C248 | M | genitalia | Maui | Makawao FR | 20.8171 | -156.2667 | 10 Aug 2002 | K. Magnacca |
|  | HYLHI009-08 | FJ411525 | C249 | F | genitalia | Maui | Makawao FR | 20.8171 | -156.2667 | 10 Aug 2002 | K. Magnacca |
|  | HYLHI010-08 | FJ411524 | C222 | F | genitalia | Moloka‘i | West Kawela Gulch | 21.1116 | -156.9072 | 2 Jun 2001 | K. Magnacca |
|  | HYLHI011-08 | FJ411523 | T202 | M | 2 legs | Moloka‘i | above W. Kawela Stream | 21.1122 | -156.9071 | 27 Aug 2005 | K. Magnacca |
|  | HYLHI012-08 | FJ411522 | T203 | M | genitalia | Moloka‘i | above W. Kawela Stream | 21.1122 | -156.9071 | 27 Aug 2005 | K. Magnacca |
|  | HYLHI013-08 | FJ411521 | T204 | M | 2 legs | Moloka‘i | above W. Kawela Stream | 21.1122 | -156.9071 | 27 Aug 2005 | K. Magnacca |
| *Hylaeus anthracinus* | HYLHI014-08 | FJ411541 | C72 | M | whole body | Hawai‘i | South Point | 18.9172 | -155.6679 | 18 Jul 1999 | K. Magnacca |
|  | HYLHI015-08 | FJ411540 | T2 | M | genitalia | Hawai‘i | Kōhanaiki | 19.6967 | -156.0460 | 5 Sep 2003 | K. Magnacca |
|  | HYLHI016-08 | FJ411539 | T59 | M | 2 legs | Hawai‘i | South Point | 18.9116 | -155.6812 | 1 Mar 2002 | K. Magnacca |
|  | HYLHI017-08 | FJ411538 | T60 | M | 2 legs | Hawai‘i | South Point | 18.9171 | -155.6647 | 1 Mar 2002 | K. Magnacca |
|  | HYLHI018-08 | FJ411537 | T62 | M | 2 legs | Kaho‘olawe | Pali O Kalapakea | 20.5572 | -156.5511 | 14 Jan 2002 | K. Wood |
|  | HYLHI019-08 | FJ411536 | C71 | M | whole body | Maui | Manawainui west | 20.6260 | -156.2083 | 23 Jun 1999 | K. Magnacca |
|  | HYLHI020-08 | FJ411535 | T61 | M | 2 legs | Maui | Manawainui west | 20.6260 | -156.2083 | 23 Jun 1999 | K. Magnacca |
|  | HYLHI021-08 | FJ411534 | C70 | M | whole body | Moloka‘i | Mo‘omomi Preserve | 21.2010 | -157.1728 | 26 Jun 1999 | K. Magnacca |
|  | HYLHI022-08 | FJ411533 | C224 | F | genitalia | Moloka‘i | Mo‘omomi Preserve | 21.2010 | -157.1728 | 4 Jun 2001 | K. Magnacca |
|  | HYLHI023-08 | FJ411532 | T11 | M | genitalia | Moloka‘i | above Ho‘olehua beach | 21.2095 | -156.9649 | 29 Aug 2005 | K. Magnacca |
|  | HYLHI024-08 | FJ411531 | T63 | M | 2 legs | Moloka‘i | Mo‘omomi Preserve | 21.2010 | -157.1728 | 2 Aug 1999 | K. Magnacca |
|  | HYLHI025-08 | FJ411530 | C69 | M | whole body | O‘ahu | Ka‘ena Point NAR | 21.5748 | -158.2800 | 12 Jun 1999 | K. Magnacca |
|  | HYLHI026-08 | FJ411529 | C142 | F | genitalia | O‘ahu | Ka‘ena Point NAR | 21.5748 | -158.2800 | 12 Jun 1999 | K. Magnacca |
|  | HYLHI027-08 | FJ411528 | T64 | F | 2 legs | O‘ahu | Ka‘ena Point NAR | 21.5748 | -158.2800 | 3 Jun 2000 | K. Magnacca |
|  | HYLHI028-08 | FJ411527 | T65 | M | 2 legs | O‘ahu | Ka‘ena Point NAR | 21.5748 | -158.2800 | 2 Mar 2002 | K. Magnacca |
| *Hylaeus assimulans* | HYLHI029-08 | FJ411545 | C96 | M | whole body | Kaho‘olawe | Kamohio | 20.5141 | -156.5908 | 17 Feb 1997 | D. Foote |
|  | HYLHI030-08 | FJ411544 | C91 | M | genitalia | Maui | Lahainaluna | 20.8792 | -156.6344 | 3 Aug 1999 | K. Magnacca |
|  | HYLHI031-08 | FJ411543 | C90 | M | genitalia | Lāna‘i | Polihua Rd. | 20.8976 | -157.0177 | 17 Jun 1999 | K. Magnacca |
|  | HYLHI032-08 | FJ411542 | T66 | M | 2 legs | Lāna‘i | Manele Rd. | 20.7564 | -156.8962 | 17 Jun 1999 | K. Magnacca |
| *Hylaeus chlorostictus* | HYLHI033-08 | FJ411550 | C59 | M | whole body | Kaua‘i | Kōke‘e Rd. | 22.1027 | -159.6774 | 24 Aug 1999 | K. Magnacca |
|  | HYLHI034-08 | FJ411549 | C138 | M | genitalia | Kaua‘i | Polihale SP | 22.0922 | -159.7485 | 25 Aug 1999 | K. Magnacca |
|  | HYLHI035-08 | FJ411548 | C185 | F | genitalia | Kaua‘i | Kukui Trail | 22.0514 | -159.6551 | 5 Jul 2000 | K. Magnacca |
|  | HYLHI036-08 | FJ411547 | C192 | F | genitalia | Kaua‘i | Pa‘aiki Valley | 22.1293 | -159.6924 | 2 Jul 2000 | K. Magnacca |
|  | HYLHI037-08 | FJ411546 | C193 | F | genitalia | Kaua‘i | Polihale SP | 22.0751 | -159.7666 | 25 Aug 1999 | K. Magnacca |
| *Hylaeus coniceps* | HYLHI038-08 | FJ411559 | C143 | M | genitalia | Hawai‘i | Saddle Rd. | 19.6727 | -155.3731 | 10 Jul 1999 | K. Magnacca |
|  | HYLHI039-08 | FJ411558 | C147 | M | whole body | Hawai‘i | Kīpuka Puaulu | 19.4400 | -155.3026 | 22 Jul 1999 | K. Magnacca |
|  | HYLHI040-08 | FJ411557 | C148 | M | whole body | Hawai‘i | Tree Molds | 19.4343 | -155.2808 | 10 Aug 1999 | K. Magnacca |
|  | HYLHI041-08 | FJ411556 | C194 | F | genitalia | Hawai‘i | Tree Molds | 19.4343 | -155.2808 | 10 Aug 1999 | K. Magnacca |
|  | HYLHI042-08 | FJ411555 | C197 | M | genitalia | Hawai‘i | KFU camp | 19.3727 | -155.8020 | 3 Aug 2000 | K. Magnacca |
|  | HYLHI043-08 | FJ411554 | C95 | M | genitalia | Maui | Ko‘olau Gap | 20.7590 | -156.2059 | 6 Aug 1999 | K. Magnacca |
|  | HYLHI044-08 | FJ411553 | C182 | M | genitalia | Maui | HALE Service Area | 20.7682 | -156.2447 | 20 Mar 2000 | R. Takumi |
|  | HYLHI045-08 | FJ411552 | C217 | F | genitalia | Maui | Pu‘u Keokea | 20.6765 | -156.3171 | 20 May 2001 | K. Magnacca |
|  | HYLHI046-08 | FJ411551 | T67 | M | 2 legs | Maui | Kaupō Trail | 20.7026 | -156.1410 | 28 Jun 2000 | K. Magnacca |
| *Hylaeus connectens* | HYLHI047-08 | FJ411571 | C32 | M | genitalia | Hawai‘i | Kīpuka Nēnē | 19.3254 | -155.2784 | 28 Jun 1998 | K. Magnacca |
|  | HYLHI048-08 | FJ411570 | C160 | F | thorax | Hawai‘i | Kīpuka Nēnē | 19.3254 | -155.2784 | 13 Jan 1999 | K. Magnacca |
|  | HYLHI049-08 | FJ411569 | C54 | M | whole body | Maui | Waihe‘e Ridge Trail | 20.9469 | -156.5472 | 5 Aug 1999 | K. Magnacca |
|  | HYLHI050-08 | FJ411568 | T51 | M | 2 legs | Maui | Pu‘u Kukui Rd. | 20.9346 | -156.6201 | 23 Jun 2000 | K. Magnacca |
|  | HYLHI051-08 | FJ411567 | C88 | M | abdomen | Lāna‘i | Munro Trail | 20.8208 | -156.8798 | 16 Jun 1999 | K. Magnacca |
|  | HYLHI052-08 | FJ411566 | C140 | M | genitalia | O‘ahu | Pu‘u Kaua | 21.4412 | -158.0944 | 29 Jul 1999 | K. Magnacca |
|  | HYLHI053-08 | FJ411565 | C226 | M | genitalia | O‘ahu | Pāhole NAR | 21.5399 | -158.1931 | 8 Jun 2001 | K. Magnacca |
|  | HYLHI054-08 | FJ411564 | T53 | F | 2 legs | O‘ahu | ‘Aiea Trail | 21.4087 | -157.8746 | 14 Aug 2000 | K. Magnacca |
|  | HYLHI055-08 | FJ411563 | C89 | M | genitalia | Kaua‘i | Kahuama‘a Flat | 22.1488 | -159.6380 | 24 Aug 1999 | K. Magnacca |
|  | HYLHI056-08 | FJ411562 | C106 | F | genitalia | Kaua‘i | Polihale SP | 22.0922 | -159.7485 | 25 Aug 1999 | K. Magnacca |
|  | HYLHI057-08 | FJ411561 | T55 | M | 2 legs | Kaua‘i | Pa‘aiki Valley | 22.1293 | -159.6924 | 2 Jul 2000 | K. Magnacca |
|  | HYLHI058-08 | FJ411560 | T56 | F | 2 legs | Kaua‘i | Pa‘aiki Valley | 22.1293 | -159.6924 | 3 Jul 2000 | K. Magnacca |
| *Hylaeus crabronoides* | HYLHI059-08 | FJ411575 | C170 | M | thorax | Hawai‘i | ‘Ōla‘a Small Tract | 19.4552 | -155.2449 | 30 Jul 2000 | K. Magnacca |
|  | HYLHI060-08 | FJ411574 | C174 | F | genitalia | Hawai‘i | KFU S. Bound. Rd. | 19.3657 | -155.8003 | 3 Aug 2000 | K. Magnacca |
|  | HYLHI061-08 | FJ411573 | T68 | M | 2 legs | Hawai‘i | KFU S. Bound. Rd. | 19.3657 | -155.8003 | 2 Aug 2000 | K. Magnacca |
|  | HYLHI062-08 | FJ411572 | T69 | M | 2 legs | Hawai‘i | Cooper Center, Volcano | 19.4339 | -155.2303 | 29 Jul 2002 | K. Magnacca |
| *Hylaeus difficilis* | HYLHI063-08 | FJ411591 | C1 | M | whole body | Hawai‘i | Mauna Loa Rd. | 19.4415 | -155.3148 | 2 Jan 1999 | K. Magnacca |
|  | HYLHI064-08 | FJ411590 | C60 | M | whole body | Hawai‘i | Ka‘alualu | 18.9676 | -155.6102 | 25 Jul 1999 | K. Magnacca |
|  | HYLHI065-08 | FJ411589 | C141 | M | genitalia | Hawai‘i | Kaloko NHP | 19.6859 | -156.0329 | 15 Aug 1999 | K. Magnacca |
|  | HYLHI066-08 | FJ411588 | C151 | M | whole body | Hawai‘i | KFU S. Bound. Rd. | 19.3657 | -155.8011 | 8 Jul 1999 | K. Magnacca |
|  | HYLHI067-08 | FJ411587 | C155 | M | whole body | Hawai‘i | Kīpuka ‘Alalā | 19.6512 | -155.6954 | 14 Jul 1999 | K. Magnacca |
|  | HYLHI068-08 | FJ411586 | C84 | M | whole body | Maui | Waikamoi Preserve | 20.7755 | -156.2275 | 4 Aug 1999 | K. Magnacca |
|  | HYLHI069-08 | FJ411585 | C85 | M | whole body | Maui | Kilohana Pali | 20.7264 | -156.2423 | 22 Jun 1999 | K. Magnacca |
|  | HYLHI070-08 | FJ411584 | C156 | M | abdomen | Maui | Waikamoi Preserve | 20.7755 | -156.2275 | 4 Aug 1999 | K. Magnacca |
|  | HYLHI071-08 | FJ411583 | C183 | M | genitalia | Maui | Pu‘u Kukui fence | 20.9362 | -156.6287 | 22 Jun 2000 | K. Magnacca |
|  | HYLHI072-08 | FJ411582 | T70 | M | 2 legs | Maui | Pu‘u Kukui Rd. | 20.9355 | -156.6261 | 22 Jun 2000 | K. Magnacca |
|  | HYLHI073-08 | FJ411581 | C86 | M | whole body | Lāna‘i | Munro Trail | 20.8255 | -156.8902 | 7 Aug 1999 | K. Magnacca |
|  | HYLHI074-08 | FJ411580 | T71 | F | 2 legs | Lāna‘i | Munro Trail | 20.8208 | -156.8798 | 16 Jun 1999 | K. Magnacca |
|  | HYLHI075-08 | FJ411579 | C50 | M | whole body | Moloka‘i | Pu‘u Kolekole | 21.1044 | -156.9025 | 28 Jun 1999 | K. Magnacca |
|  | HYLHI076-08 | FJ411578 | T72 | M | 2 legs | Moloka‘i | Kamakou Rd. | 21.1243 | -156.9183 | 27 Jun 1999 | K. Magnacca |
|  | HYLHI077-08 | FJ411577 | T73 | M | 2 legs | Moloka‘i | Pu‘u Kolekole Rd. | 21.1075 | -156.8969 | 2 Jun 2001 | K. Magnacca |
|  | HYLHI078-08 | FJ411576 | T74 | M | 2 legs | Moloka‘i | Pu‘u Kolekole Rd. | 21.1075 | -156.8969 | 2 Jun 2001 | K. Magnacca |
| *Hylaeus dimidiatus* | HYLHI079-08 | FJ411594 | C237 | M | genitalia | Hawai‘i | Kīpuka ‘Alalā | 19.6512 | -155.6954 | 14 Jul 1999 | K. Magnacca |
|  | HYLHI080-08 | FJ411593 | C242 | F | genitalia | Hawai‘i | Pu‘u Wa‘awa‘a WS | 19.7362 | -155.8402 | 1 Aug 2002 | K. Magnacca |
|  | HYLHI081-08 | FJ411592 | T75 | M | 2 legs | Hawai‘i | Pu‘u Wa‘awa‘a WS | 19.7362 | -155.8402 | 1 Aug 2002 | K. Magnacca |
| *Hylaeus dumetorum* | HYLHI082-08 | FJ411600 | C7 | M | whole body | Hawai‘i | Tree Planting Rd. | 19.6647 | -155.2786 | 5 Jan 1999 | K. Magnacca |
|  | HYLHI083-08 | FJ411599 | C93 | F | genitalia | Hawai‘i | KFU S. Bound. Rd. | 19.3657 | -155.8011 | 8 Jul 1999 | K. Magnacca |
|  | HYLHI084-08 | FJ411598 | C146 | F | genitalia | Hawai‘i | Cymbidium Acres | 19.4474 | -155.2376 | 10 Dec 1996 | K. Magnacca |
|  | HYLHI085-08 | FJ411597 | C169 | M | genitalia | Hawai‘i | Halemaumau Trail | 19.4281 | -155.2594 | 29 Jul 2000 | K. Magnacca |
|  | HYLHI086-08 | FJ411596 | T13 | M | genitalia | Hawai‘i | Kahuku | 19.1179 | -155.6755 | 1 Feb 2006 | K. Magnacca |
|  | HYLHI087-08 | FJ411595 | T76 | M | 2 legs | Hawai‘i | Mountain House Rd. | 19.1361 | -155.6131 | 25 May 2001 | K. Magnacca |
| *Hylaeus facilis* | HYLHI088-08 | FJ411601 | T206 | M | 2 legs | Moloka‘i | Kuololimu | 21.1869 | -156.9495 | 3 Sep 2005 | K. Magnacca |
| *Hylaeus filicum* | HYLHI089-08 | FJ411603 | T197 | F | 2 legs | Hawai‘i | KFU S. Bound. Rd. | 19.3657 | -155.8003 | 3 Aug 2000 | K. Magnacca |
|  | HYLHI090-08 | FJ411602 | C243 | M | genitalia | Hawai‘i | Pu‘u Wa‘awa‘a WS | 19.7362 | -155.8402 | 1 Aug 2002 | K. Magnacca |
| *Hylaeus flavifrons* | HYLHI091-08 | FJ411607 | C19 | M | whole body | Kaua‘i | Polihale | 22.0751 | -159.7666 | 17 Jan 1999 | K. Magnacca |
|  | HYLHI092-08 | FJ411606 | C33 | M | genitalia | Kaua‘i | Polihale | 22.0751 | -159.7666 | 17 Jan 1999 | K. Magnacca |
|  | HYLHI093-08 | FJ411605 | C229 | F | genitalia | Kaua‘i | Polihale | 22.0751 | -159.7666 | 25 Aug 1999 | K. Magnacca |
|  | HYLHI094-08 | FJ411604 | C240 | M | genitalia | Ni‘ihau | Lehua, West Horn | 22.0258 | -160.1008 | 19 Feb 2002 | K. Wood |
| *Hylaeus flavipes* | HYLHI095-08 | FJ411616 | C3 | M | whole body | Hawai‘i | Kīpuka Nēnē | 19.3271 | -155.2791 | 3 Jan 1999 | K. Magnacca |
|  | HYLHI096-08 | FJ411615 | C15 | M | whole body | Hawai‘i | Kīpuka Nēnē | 19.3271 | -155.2791 | 5 Jul 1998 | K. Magnacca |
|  | HYLHI097-08 | FJ411614 | C135 | M | genitalia | Hawai‘i | Pōhakuloa, Mana‘o | 19.8100 | -155.5900 | 19 May 1995 | P. Oboyski |
|  | HYLHI098-08 | FJ411613 | C136 | M | genitalia | Hawai‘i | Hale Pōhaku | 19.7623 | -155.4565 | 10 Jul 1999 | K. Magnacca |
|  | HYLHI099-08 | FJ411612 | C219 | M | genitalia | Hawai‘i | South Point | 18.9116 | -155.6812 | 28 May 2001 | K. Magnacca |
|  | HYLHI100-08 | FJ411611 | C65 | M | whole body | Lāna‘i | Kahue | 20.8833 | -156.9628 | 7 Aug 1999 | K. Magnacca |
|  | HYLHI101-08 | FJ411610 | C206 | M | genitalia | Lāna‘i | Kahue | 20.8833 | -156.9628 | 7 Aug 1999 | K. Magnacca |
|  | HYLHI102-08 | FJ411609 | T77 | M | 2 legs | Lāna‘i | Kahue | 20.8833 | -156.9628 | 24 Jun 2000 | K. Magnacca |
|  | HYLHI103-08 | FJ411608 | T78 | M | 2 legs | Lāna‘i | Kahue | 20.8735 | -156.9655 | 6 Jun 2001 | K. Magnacca |
| *Hylaeus fuscipennis* | HYLHI104-08 | FJ411625 | C164 | M | whole body | Maui | Pu‘u Kukui fence | 20.9362 | -156.6287 | 22 Jun 2000 | K. Magnacca |
|  | HYLHI105-08 | FJ411624 | T107 | F | thorax | Maui | Ka‘ulalewelewe | 20.9345 | -156.6162 | 10 Aug 2000 | K. Magnacca |
|  | HYLHI106-08 | FJ411623 | T108 | M | thorax | Maui | Kahoma | 20.9041 | -156.6292 | 22 May 2001 | K. Magnacca |
|  | HYLHI107-08 | FJ411622 | C83 | M | genitalia | Lāna‘i | Munro Trail | 20.8208 | -156.8798 | 16 Jun 1999 | K. Magnacca |
|  | HYLHI108-08 | FJ411621 | C225 | M | genitalia | Lāna‘i | Kaiholena Ridge | 20.8241 | -156.8946 | 7 Jun 2001 | K. Magnacca |
|  | HYLHI109-08 | FJ411620 | C40 | M | legs | Moloka‘i | Kamakou Rd. | 21.1155 | -156.9175 | 7 Jun 1996 | K. Magnacca |
|  | HYLHI110-08 | FJ411619 | C82 | M | genitalia | Moloka‘i | Kamakou Rd. | 21.1243 | -156.9183 | 27 Jun 1999 | K. Magnacca |
|  | HYLHI111-08 | FJ411618 | C223 | M | genitalia | Moloka‘i | East Kawela Gulch | 21.1108 | -156.9057 | 2 Jun 2001 | K. Magnacca |
|  | HYLHI112-08 | FJ411617 | T8 | M | hind leg | Moloka‘i | above W. Kawela Stream | 21.1122 | -156.9071 | 27 Aug 2005 | K. Magnacca |
| *Hylaeus haleakalae* | HYLHI113-08 | FJ411635 | C177 | M | genitalia | Maui | Pu‘u Kukui Tr. 4500m | 20.9212 | -156.5994 | 8 Aug 2000 | K. Magnacca |
|  | HYLHI114-08 | FJ411634 | C191 | F | genitalia | Maui | Pu‘u Kukui Tr. 5000m | 20.9289 | -156.6088 | 11 Aug 2000 | K. Magnacca |
|  | HYLHI115-08 | FJ411633 | C250 | M | genitalia | Maui | Makawao FR | 20.8074 | -156.2530 | 10 Aug 2002 | K. Magnacca |
|  | HYLHI116-08 | FJ411632 | T79 | F | 2 legs | Maui | Makawao FR | 20.8171 | -156.2667 | 10 Aug 2002 | K. Magnacca |
|  | HYLHI117-08 | FJ411631 | C77 | M | genitalia | Moloka‘i | West Kawela Gulch | 21.1117 | -156.9069 | 28 Jun 1999 | K. Magnacca |
|  | HYLHI118-08 | FJ411630 | C78 | F | genitalia | Moloka‘i | Kamakou Rd. | 21.1243 | -156.9183 | 27 Jun 1999 | K. Magnacca |
|  | HYLHI119-08 | FJ411629 | T9 | M | genitalia | Moloka‘i | above W. Kawela Stream | 21.1122 | -156.9071 | 27 Aug 2005 | K. Magnacca |
|  | HYLHI120-08 | FJ411628 | T141 | F | 2 legs | Moloka‘i | Kawela Rd. | 21.1075 | -156.8969 | 2 Jun 2001 | K. Magnacca |
|  | HYLHI121-08 | FJ411627 | T142 | M | 2 legs | Moloka‘i | East Kawela Gulch | 21.1108 | -156.9057 | 2 Jun 2001 | K. Magnacca |
| *Hylaeus hilaris* | HYLHI122-08 | FJ411636 | C87 | M | single leg | Moloka‘i | Mo‘omomi Preserve | 21.2010 | -157.1728 | 30 Jun 1999 | K. Magnacca |
| *Hylaeus hirsutulus* | HYLHI123-08 | FJ411640 | C161 | M | abdomen | Kaua‘i | Alaka‘i, 1.5 mi. NW Keaku | 22.0731 | -159.5386 | 2 Nov 1999 | D. Hopper |
|  | HYLHI124-08 | FJ411639 | C162 | F | abdomen | Kaua‘i | Alaka‘i, 1.5 mi. NW Keaku | 22.0731 | -159.5386 | 2 Nov 1999 | D. Hopper |
|  | HYLHI125-08 | FJ411638 | T80 | M | 2 legs | Kaua‘i | Nu‘alolo Cliff Trail | 22.1478 | -159.6833 | 3 Jul 2000 | K. Magnacca |
|  | HYLHI126-08 | FJ411637 | T81 | M | 2 legs | Kaua‘i | Mōhihi Trail | 22.1098 | -159.5936 | 8 Jul 2000 | K. Magnacca |
| *Hylaeus hostilis* | HYLHI127-08 | FJ411643 | C58 | M | whole body | Kaua‘i | Polihale SP | 22.0751 | -159.7666 | 25 Aug 1999 | K. Magnacca |
|  | HYLHI128-08 | FJ411642 | T82 | F | 2 legs | Kaua‘i | Nu‘alolo Cliff Trail | 22.1478 | -159.6833 | 3 Jul 2000 | K. Magnacca |
|  | HYLHI129-08 | FJ411641 | T83 | F | 2 legs | Kaua‘i | ‘Awa‘awapuhi Trail | 22.1482 | -159.6659 | 4 Jul 2000 | K. Magnacca |
| *Hylaeus hula* | HYLHI130-08 | FJ411647 | C103 | M | whole body | Hawai‘i | Tree Molds | 19.4343 | -155.2808 | 10 Aug 1999 | K. Magnacca |
|  | HYLHI131-08 | FJ411646 | C207 | M | genitalia | Hawai‘i | Tree Molds | 19.4343 | -155.2808 | 10 Aug 1999 | K. Magnacca |
|  | HYLHI132-08 | FJ411645 | C244 | F | genitalia | Hawai‘i | Pu‘u Wa‘awa‘a WS | 19.7362 | -155.8402 | 1 Aug 2002 | K. Magnacca |
|  | HYLHI133-08 | FJ411644 | T84 | M | 2 legs | Hawai‘i | HAVO boundary fence | 19.4482 | -155.2898 | 26 Jul 2002 | K. Magnacca |
| *Hylaeus inquilina* | HYLHI134-08 | FJ411649 | C5 | M | whole body | Hawai‘i | 0.9 mi. S of Mauna Loa Rd. | 19.4723 | -155.3850 | 4 Jan 1999 | K. Magnacca |
|  | HYLHI135-08 | FJ411648 | T85 | M | 2 legs | Hawai‘i | near Tree Molds | 19.4344 | -155.2855 | 11 Jun 2000 | K. Magnacca |
| *Hylaeus kauaiensis* | HYLHI136-08 | FJ411654 | C73 | M | whole body | Kaua‘i | Alaka‘i Swamp Trail | 22.1362 | -159.6256 | 3 Jul 1999 | K. Magnacca |
|  | HYLHI137-08 | FJ411653 | T89 | M | 2 legs | Kaua‘i | Alaka‘i Swamp Trail | 22.1362 | -159.6256 | 21 Aug 1999 | K. Magnacca |
|  | HYLHI138-08 | FJ411652 | T90 | M | 2 legs | Kaua‘i | Mōhihi Trail | 22.1133 | -159.5872 | 8 Jul 2000 | K. Magnacca |
|  | HYLHI139-08 | FJ411651 | T144 | F | soaking body | Kaua‘i | Mt. Kahili | 21.9720 | -159.4995 | 1 Oct 1997 | A. Asquith |
| *Hylaeus kokeensis* | HYLHI140-08 | FJ411658 | C104 | F | genitalia | Kaua‘i | Kōke‘e Rd. | 22.1027 | -159.6774 | 24 Aug 1999 | K. Magnacca |
|  | HYLHI141-08 | FJ411657 | C167 | M | whole body | Kaua‘i | ‘Awa‘awapuhi Trail | 22.1482 | -159.6659 | 4 Jul 2000 | K. Magnacca |
|  | HYLHI142-08 | FJ411656 | T91 | F | 2 legs | Kaua‘i | ‘Awa‘awapuhi Trail | 22.1482 | -159.6659 | 3 Jul 2000 | K. Magnacca |
|  | HYLHI143-08 | FJ411655 | T92 | F | 2 legs | Kaua‘i | ‘Awa‘awapuhi Trail | 22.1482 | -159.6659 | 8 Jul 2000 | K. Magnacca |
| *Hylaeus kona* | HYLHI144-08 | FJ411660 | C67 | M | whole body | Hawai‘i | Kīpuka ‘Alalā | 19.6512 | -155.6954 | 14 Jul 1999 | K. Magnacca |
|  | HYLHI145-08 | FJ411659 | T93 | M | 2 legs | Hawai‘i | Kīpuka ‘Alalā | 19.6512 | -155.6954 | 14 Jul 1999 | K. Magnacca |
| *Hylaeus kuakea* | HYLHI146-08 | FJ411661 | C181 | M | whole body | O‘ahu | Moho Gulch Ridge | 21.4698 | -158.0989 | 1 Aug 1997 | D. Hopper |
| *Hylaeus kukui* | HYLHI147-08 | FJ411664 | T5 | F | 2 legs | Hawai‘i | Kahuku, nr. reservoir | 19.1059 | -155.6720 | 1 Aug 2005 | K. Magnacca |
|  | HYLHI148-08 | FJ411663 | T199 | M | 2 legs | Hawai‘i | Kahuku | 19.1179 | -155.6755 | 10 Jul 2005 | K. Magnacca |
|  | HYLHI149-08 | FJ411662 | C180 | M | genitalia | Maui | Pu‘u Kukui Tr. 7000m | 20.9335 | -156.6130 | 11 Aug 2000 | K. Magnacca |
| *Hylaeus laetus* | HYLHI150-08 | FJ411680 | C2 | M | whole body | Hawai‘i | Kīpuka Nēnē | 19.3271 | -155.2791 | 3 Jan 1999 | K. Magnacca |
|  | HYLHI151-08 | FJ411679 | C11 | M | whole body | Hawai‘i | Kīpuka Nēnē | 19.3254 | -155.2784 | 13 Jan 1999 | K. Magnacca |
|  | HYLHI152-08 | FJ411678 | C16 | F | whole body | Hawai‘i | Kīpuka Nēnē | 19.3254 | -155.2784 | 13 Jan 1999 | K. Magnacca |
|  | HYLHI153-08 | FJ411677 | T94 | M | 2 legs | Hawai‘i | Kīpuka Kalawamana | 19.7339 | -155.6657 | 13 Jul 1999 | K. Magnacca |
|  | HYLHI154-08 | FJ411676 | T95 | M | 2 legs | Hawai‘i | ‘Ahumoa | 19.8134 | -155.6261 | 15 Aug 1999 | K. Magnacca |
|  | HYLHI155-08 | FJ411675 | C49 | M | whole body | Maui | Lahainaluna | 20.8792 | -156.6344 | 3 Aug 1999 | K. Magnacca |
|  | HYLHI156-08 | FJ411674 | T96 | M | 2 legs | Maui | Lahainaluna | 20.8792 | -156.6344 | 3 Aug 1999 | K. Magnacca |
|  | HYLHI157-08 | FJ411673 | T97 | F | 2 legs | Maui | Kahoma | 20.9027 | -156.6387 | 23 May 2001 | K. Magnacca |
|  | HYLHI158-08 | FJ411672 | C51 | M | whole body | Lāna‘i | Kanepu‘u Preserve, Kahue | 20.8735 | -156.9655 | 15 Jun 1999 | K. Magnacca |
|  | HYLHI159-08 | FJ411671 | T98 | M | 2 legs | Lāna‘i | Kahue | 20.8833 | -156.9628 | 24 Jun 2000 | K. Magnacca |
|  | HYLHI160-08 | FJ411670 | C227 | F | genitalia | O‘ahu | Pāhole NAR | 21.5441 | -158.1945 | 8 Jun 2001 | K. Magnacca |
|  | HYLHI161-08 | FJ411669 | C4 | M | whole body | Kaua‘i | Nu‘alolo Trail | 22.1401 | -159.6757 | 19 Jan 1999 | K. Magnacca |
|  | HYLHI162-08 | FJ411668 | T99 | M | genitalia | Kaua‘i | Black Pipe Trail | 22.1142 | -159.6641 | 2 Jul 1999 | K. Magnacca |
|  | HYLHI163-08 | FJ411667 | T100 | M | 2 legs | Kaua‘i | Pa‘aiki Valley | 22.1293 | -159.6924 | 2 Jul 2000 | K. Magnacca |
|  | HYLHI164-08 | FJ411666 | T101 | F | genitalia | Kaua‘i | ‘Awa‘awapuhi Trail | 22.1482 | -159.6659 | 3 Jul 2000 | K. Magnacca |
|  | HYLHI165-08 | FJ411665 | T102 | M | 2 legs | Kaua‘i | Mōhihi Trail | 22.1098 | -159.5936 | 8 Jul 2000 | K. Magnacca |
| *Hylaeus longiceps* | HYLHI166-08 | FJ411691 | C64 | M | whole body | Maui | Wai‘ehu dune | 20.9241 | -156.4950 | 5 Aug 1999 | K. Magnacca |
|  | HYLHI167-08 | FJ411690 | T111 | M | 2 legs | Maui | Wai‘ehu dune | 20.9241 | -156.4950 | 21 May 2001 | K. Magnacca |
|  | HYLHI168-08 | FJ411689 | C63 | M | whole body | Lāna‘i | Polihua Rd. | 20.8976 | -157.0177 | 17 Jun 1999 | K. Magnacca |
|  | HYLHI169-08 | FJ411688 | T112 | M | 2 legs | Lāna‘i | Shipwreck Beach | 20.9144 | -156.9006 | 6 Jun 2001 | K. Magnacca |
|  | HYLHI170-08 | FJ411687 | C62 | M | whole body | Moloka‘i | Mo‘omomi Preserve | 21.2010 | -157.1728 | 26 Jun 1999 | K. Magnacca |
|  | HYLHI171-08 | FJ411686 | T113 | F | 2 legs | Moloka‘i | Mo‘omomi Preserve | 21.2010 | -157.1728 | 26 Jun 1999 | K. Magnacca |
|  | HYLHI172-08 | FJ411685 | T114 | M | 2 legs | Moloka‘i | Mo‘omomi Preserve | 21.2010 | -157.1728 | 2 Aug 1999 | K. Magnacca |
|  | HYLHI173-08 | FJ411684 | C10 | F | whole body | O‘ahu | Ka‘ena Point NAR | 21.5748 | -158.2800 | 31 Dec 1998 | K. Magnacca |
|  | HYLHI174-08 | FJ411683 | C61 | M | whole body | O‘ahu | Ka‘ena Point NAR | 21.5748 | -158.2800 | 12 Jun 1999 | K. Magnacca |
|  | HYLHI175-08 | FJ411682 | T115 | M | 2 legs | O‘ahu | Ka‘ena Point NAR | 21.5756 | -158.2709 | 2 Mar 2002 | K. Magnacca |
|  | HYLHI176-08 | FJ411681 | T116 | F | genitalia | O‘ahu | Ka‘ena Point NAR | 21.5748 | -158.2800 | 2 Mar 2002 | K. Magnacca |
| *Hylaeus mana* | HYLHI177-08 | FJ411692 | C239 | M | genitalia | O‘ahu | Manana Trail | 21.4440 | -157.9057 | 3 Mar 2002 | K. Magnacca |
| *Hylaeus mimicus* | HYLHI178-08 | FJ411695 | C101 | M | genitalia | O‘ahu | Wiliwilinui Trail | 21.3236 | -157.7561 | 26 Jul 1999 | K. Magnacca |
|  | HYLHI179-08 | FJ411694 | T117 | M | 2 legs | O‘ahu | ‘Aiea Trail | 21.4087 | -157.8746 | 2 Jun 2000 | K. Magnacca |
|  | HYLHI180-08 | FJ411693 | T118 | F | 2 legs | O‘ahu | ‘Aiea Trail | 21.4147 | -157.8636 | 14 Aug 2000 | K. Magnacca |
| *Hylaeus muranus* | HYLHI181-08 | FJ411698 | T86 | F | 2 legs | Hawai‘i | Old Jap. School, Volcano | 19.4338 | -155.2394 | 6 Aug 2000 | K. Magnacca |
|  | HYLHI182-08 | FJ411697 | T140 | F | 2 legs | Hawai‘i | Old Jap. School, Volcano | 19.4338 | -155.2394 | 5 Aug 2000 | K. Magnacca |
|  | HYLHI183-08 | FJ411696 | T192 | F | gut & ovaries | Hawai‘i | Old Jap. School, Volcano | 19.4338 | -155.2394 | 29 Jul 2002 | K. Magnacca |
| *Hylaeus mutatus* | HYLHI184-08 | FJ411703 | C105 | M | genitalia | Kaua‘i | Kahuama‘a Flat | 22.1488 | -159.6380 | 24 Aug 1999 | K. Magnacca |
|  | HYLHI185-08 | FJ411702 | C184 | M | genitalia | Kaua‘i | ‘Awa‘awapuhi Trail | 22.1482 | -159.6659 | 4 Jul 2000 | K. Magnacca |
|  | HYLHI186-08 | FJ411701 | C186 | F | genitalia | Kaua‘i | ‘Awa‘awapuhi Trail | 22.1482 | -159.6659 | 4 Jul 2000 | K. Magnacca |
|  | HYLHI187-08 | FJ411700 | C195 | F | genitalia | Kaua‘i | ‘Awa‘awapuhi Trail | 22.1482 | -159.6659 | 3 Jul 2000 | K. Magnacca |
|  | HYLHI188-08 | FJ411699 | T145 | M | 2 legs | Kaua‘i | Kuia Valley | 22.1348 | -159.6935 | 7 Jul 2000 | K. Magnacca |
| *Hylaeus nivicola* | HYLHI189-08 | FJ411710 | C149 | M | whole body | Maui | Halemau‘u Trail | 20.7524 | -156.2286 | 19 Jun 1999 | K. Magnacca |
|  | HYLHI190-08 | FJ411709 | T120 | M | 2 legs | Maui | Pu‘u Keokea | 20.6765 | -156.3171 | 20 May 2001 | K. Magnacca |
|  | HYLHI191-08 | FJ411708 | T121 | M | 2 legs | Maui | Pu‘u Keokea | 20.6765 | -156.3171 | 20 May 2001 | K. Magnacca |
| *Hylaeus ombrias* | HYLHI192-08 | FJ411716 | C66 | M | whole body | Hawai‘i | South Point | 18.9116 | -155.6812 | 17 Jul 1999 | K. Magnacca |
|  | HYLHI193-08 | FJ411715 | T122 | M | 2 legs | Hawai‘i | Kīpuka Kalawamana | 19.7339 | -155.6657 | 13 Jul 1999 | K. Magnacca |
|  | HYLHI194-08 | FJ411714 | T123 | M | 2 legs | Hawai‘i | ‘Ahumoa | 19.8134 | -155.6261 | 15 Aug 1999 | K. Magnacca |
|  | HYLHI195-08 | FJ411713 | T124 | F | genitalia | Hawai‘i | South Point | 18.9172 | -155.6679 | 1 Mar 2002 | K. Magnacca |
|  | HYLHI196-08 | FJ411712 | T146 | F | 2 legs | Hawai‘i | South Point | 18.9116 | -155.6812 | 1 Mar 2002 | K. Magnacca |
| *Hylaeus paradoxicus* | HYLHI197-08 | FJ411720 | C81 | M | genitalia | Hawai‘i | Kīpuka ‘Alalā | 19.6512 | -155.6954 | 14 Jul 1999 | K. Magnacca |
|  | HYLHI198-08 | FJ411719 | T125 | M | 2 legs | Hawai‘i | Pu‘u Wa‘awa‘a WS | 19.7362 | -155.8402 | 1 Aug 2002 | K. Magnacca |
| *Hylaeus pele* | HYLHI199-08 | FJ411725 | C13 | M | whole body | Hawai‘i | Mauna Loa Rd. | 19.4405 | -155.3071 | 2 Jan 1999 | K. Magnacca |
|  | HYLHI200-08 | FJ411724 | C43 | M | genitalia | Hawai‘i | Kīpuka Nēnē | 19.3254 | -155.2784 | 4 Jan 1999 | K. Magnacca |
|  | HYLHI201-08 | FJ411723 | C68 | M | whole body | Hawai‘i | Kīpuka ‘Alalā | 19.6512 | -155.6954 | 14 Jul 1999 | K. Magnacca |
|  | HYLHI202-08 | FJ411722 | C134 | M | genitalia | Hawai‘i | Mauna Loa Strip Road | 19.4405 | -155.3071 | 2 Jan 1999 | K. Magnacca |
|  | HYLHI203-08 | FJ411721 | C137 | M | genitalia | Hawai‘i | Kīpuka Puaulu | 19.4400 | -155.3026 | 22 Jul 1999 | K. Magnacca |
| *Hylaeus psammobius* | HYLHI204-08 | FJ411727 | C246 | M | genitalia | Maui | Eleilei Bay | 20.9431 | -156.3299 | 8 Aug 2002 | K. Magnacca |
|  | HYLHI205-08 | FJ411726 | C247 | F | genitalia | Maui | Eleilei Bay | 20.9431 | -156.3299 | 8 Aug 2002 | K. Magnacca |
| *Hylaeus pubescens* | HYLHI206-08 | FJ411732 | C14 | M | whole body | Hawai‘i | Devastation Trail | 19.4074 | -155.2536 | 8 Jan 1999 | K. Magnacca |
|  | HYLHI207-08 | FJ411731 | C23 | M | abdomen | Hawai‘i | Devastation Trail | 19.4074 | -155.2536 | 8 Jan 1999 | K. Magnacca |
|  | HYLHI208-08 | FJ411730 | C221 | M | genitalia | Hawai‘i | Upper Hamakua Ditch | 20.0577 | -155.6679 | 29 May 2001 | K. Magnacca |
|  | HYLHI209-08 | FJ411729 | T126 | M | 2 legs | Hawai‘i | Mountain House Rd. | 19.1361 | -155.6131 | 25 May 2001 | K. Magnacca |
|  | HYLHI210-08 | FJ411728 | T181 | F | thorax | Hawai‘i | Kona Forest Unit camp | 19.3727 | -155.8020 | 2 Aug 2000 | K. Magnacca |
| *Hylaeus rugulosus* | HYLHI211-08 | FJ411735 | C241 | F | genitalia | Hawai‘i | Nāpau Trail | 19.3706 | -155.1536 | 14 Jul 2002 | K. Magnacca |
| *Hylaeus setosifrons* | HYLHI212-08 | FJ411739 | C80 | M | genitalia | Hawai‘i | Tree Molds | 19.4343 | -155.2808 | 9 Aug 1999 | K. Magnacca |
|  | HYLHI213-08 | FJ411738 | T128 | M | 2 legs | Hawai‘i | Earthquake Trail | 19.4253 | -155.2573 | 9 Aug 1999 | K. Magnacca |
|  | HYLHI214-08 | FJ411737 | T130 | M | 2 legs | Hawai‘i | Kīpuka Puaulu | 19.4400 | -155.3026 | 31 Jul 2002 | K. Magnacca |
|  | HYLHI215-08 | FJ411736 | T185 | F | genitalia | Hawai‘i | Old Jap. School, Volcano | 19.4338 | -155.2394 | 5 Aug 2000 | K. Magnacca |
| *Hylaeus solaris* | HYLHI216-08 | FJ411742 | C100 | M | whole body | Kaua‘i | Polihale SP | 22.0751 | -159.7666 | 25 Aug 1999 | K. Magnacca |
|  | HYLHI217-08 | FJ411741 | C150 | M | whole body | Kaua‘i | Polihale SP | 22.0751 | -159.7666 | 12 Jun 1999 | K. Magnacca |
|  | HYLHI218-08 | FJ411740 | C230 | F | genitalia | Kaua‘i | Polihale | 22.0751 | -159.7666 | 25 Aug 1999 | K. Magnacca |
| *Hylaeus specularis* | HYLHI219-08 | FJ411756 | C92 | M | genitalia | Hawai‘i | KFU S. Bound. Rd. | 19.3657 | -155.8011 | 8 Jul 1999 | K. Magnacca |
|  | HYLHI220-08 | FJ411755 | C171 | M | genitalia | Hawai‘i | KFU S. Bound. Rd. | 19.3657 | -155.8003 | 2 Aug 2000 | K. Magnacca |
|  | HYLHI221-08 | FJ411754 | C173 | F | genitalia | Hawai‘i | KFU S. Bound. Rd. | 19.3657 | -155.8003 | 3 Aug 2000 | K. Magnacca |
|  | HYLHI222-08 | FJ411753 | T205 | M | 2 legs | Moloka‘i | above W. Kawela Stream | 21.1122 | -156.9071 | 27 Aug 2005 | K. Magnacca |
|  | HYLHI223-08 | FJ411752 | C238 | F | genitalia | O‘ahu | Manana Trail | 21.4461 | -157.9023 | 19 Feb 2002 | K. Magnacca |
|  | HYLHI224-08 | FJ411751 | C99 | M | genitalia | Kaua‘i | Nā Pali-Kona FR | 22.1434 | -159.6571 | 4 Jul 1999 | K. Magnacca |
|  | HYLHI225-08 | FJ411750 | C168 | F | genitalia | Kaua‘i | ‘Awa‘awapuhi Trail | 22.1434 | -159.6571 | 4 Jul 2000 | K. Magnacca |
|  | HYLHI226-08 | FJ411749 | T131 | M | genitalia | Kaua‘i | ‘Awa‘awapuhi Trail | 22.1434 | -159.6571 | 5 Jul 2000 | K. Magnacca |
| *Hylaeus sphecodoides* | HYLHI227-08 | FJ411761 | C57 | M | whole body | Hawai‘i | Kīpuka Nēnē | 19.3275 | -155.2789 | 11 Jul 1999 | K. Magnacca |
|  | HYLHI228-08 | FJ411760 | C233 | M | whole body | Hawai‘i | Kona Forest Unit 3613 | 19.3709 | -155.8358 |  | D. Foote |
|  | HYLHI229-08 | FJ411759 | T132 | F | 2 legs | Hawai‘i | Kīpuka ‘Alalā | 19.6404 | -155.6876 | 13 Jul 1999 | K. Magnacca |
|  | HYLHI230-08 | FJ411758 | T133 | F | 2 legs | Hawai‘i | South Point | 18.9116 | -155.6812 | 1 Mar 2002 | K. Magnacca |
|  | HYLHI231-08 | FJ411757 | T134 | M | 2 legs | Hawai‘i | Keauhou Ranch | 19.4819 | -155.2981 | 29 Jun 2002 | K. Magnacca |
| *Hylaeus takumiae* | HYLHI232-08 | FJ411762 | C97 | M | abdomen | Maui | Kilohana Pali | 20.7264 | -156.2423 | 27 Apr 1999 | R. Takumi |
| *Hylaeus unicus* | HYLHI233-08 | FJ411775 | C165 | M | whole body | Maui | Pu‘u Kukui Rd. | 20.9355 | -156.6261 | 22 Jun 2000 | K. Magnacca |
|  | HYLHI234-08 | FJ411774 | C178 | M | genitalia | Maui | Pu‘u Kukui Tr. 4500m | 20.9212 | -156.5994 | 8 Aug 2000 | K. Magnacca |
|  | HYLHI235-08 | FJ411773 | C179 | F | genitalia | Maui | Pu‘u Kukui Tr. 4500m | 20.9212 | -156.5994 | 8 Aug 2000 | K. Magnacca |
|  | HYLHI236-08 | FJ411772 | T41 | M | right legs | Maui | Makawao FR | 20.8074 | -156.2530 | 10 Aug 2002 | K. Magnacca |
|  | HYLHI237-08 | FJ411771 | C75 | M | whole body | Lāna‘i | Munro Trail | 20.8208 | -156.8798 | 16 Jun 1999 | K. Magnacca |
|  | HYLHI238-08 | FJ411770 | C76 | M | genitalia | Moloka‘i | Kamakou Rd. | 21.1243 | -156.9183 | 27 Jun 1999 | K. Magnacca |
|  | HYLHI239-08 | FJ411769 | T6 | M | genitalia | Moloka‘i | above W. Kawela Stream | 21.1122 | -156.9071 | 27 Aug 2005 | K. Magnacca |
|  | HYLHI240-08 | FJ411768 | T42 | M | soaking body | Moloka‘i | Pepeopae boardwalk | 21.1188 | -156.9002 | 2 Jun 2001 | K. Magnacca |
|  | HYLHI241-08 | FJ411767 | C74 | M | whole body | O‘ahu | Wiliwilinui Trail | 21.3236 | -157.7561 | 13 Jun 1999 | K. Magnacca |
|  | HYLHI242-08 | FJ411766 | C139 | M | genitalia | O‘ahu | Pu‘u Kaua | 21.4412 | -158.0944 | 29 Jul 1999 | K. Magnacca |
|  | HYLHI243-08 | FJ411765 | T43 | M | right legs | O‘ahu | ‘Aiea Trail | 21.4147 | -157.8636 | 14 Aug 2000 | K. Magnacca |
|  | HYLHI244-08 | FJ411764 | T44 | M | genitalia | O‘ahu | Manana Trail | 21.4487 | -157.8928 | 19 Feb 2002 | K. Magnacca |
|  | HYLHI245-08 | FJ411763 | T214 | F | thoracic muscles & eggs | O‘ahu | Manana Trail | 21.4461 | -157.9023 | 19 Feb 2002 | K. Magnacca |
| *Hylaeus volatilis* | HYLHI246-08 | FJ411781 | C55 | M | whole body | Maui | Halemau‘u Trail | 20.7558 | -156.2207 | 23 Jun 1999 | K. Magnacca |
|  | HYLHI247-08 | FJ411780 | C56 | M | whole body | Maui | Halemau‘u Trail | 20.7558 | -156.2207 | 23 Jun 1999 | K. Magnacca |
|  | HYLHI248-08 | FJ411779 | C163 | M | genitalia | Maui | HALE Service Area | 20.7682 | -156.2447 | 20 Mar 2000 | R. Takumi |
|  | HYLHI249-08 | FJ411778 | T147 | M | 2 legs | Maui | Lau‘ulu Trail | 20.7314 | -156.1481 | 27 Jun 2000 | K. Magnacca |
|  | HYLHI250-08 | FJ411777 | T148 | M | 2 legs | Maui | Pu‘u Keokea | 20.6765 | -156.3171 | 20 May 2001 | K. Magnacca |
|  | HYLHI251-08 | FJ411776 | C94 | M | genitalia | Maui | Lahainaluna | 20.8792 | -156.6344 | 3 Aug 1999 | K. Magnacca |
| *Hylaeus volcanicus* | HYLHI252-08 | FJ411791 | C6 | M | whole body | Hawai‘i | Mauna Loa Rd. | 19.4415 | -155.3148 | 2 Jan 1999 | K. Magnacca |
|  | HYLHI253-08 | FJ411790 | C20 | M | abdomen | Hawai‘i | 0.9 mi. S of Mauna Loa Rd. | 19.4723 | -155.3850 | 4 Jan 1999 | K. Magnacca |
|  | HYLHI254-08 | FJ411789 | C21 | M | abdomen | Hawai‘i | 0.9 mi. S of Mauna Loa Rd. | 19.4723 | -155.3850 | 4 Jan 1999 | K. Magnacca |
|  | HYLHI255-08 | FJ411788 | C187 | F | genitalia | Hawai‘i | Crater Rim Trail | 19.4245 | -155.2924 | 24 Jul 2000 | K. Magnacca |
|  | HYLHI256-08 | FJ411787 | T45 | M | soaking body | Hawai‘i | Pu‘u Wa‘awa‘a WS | 19.7175 | -155.8836 | 30 May 2001 | K. Magnacca |
|  | HYLHI257-08 | FJ411786 | C166 | M | whole body | Maui | Kaupō Trail | 20.6930 | -156.1390 | 28 Jun 2000 | K. Magnacca |
|  | HYLHI258-08 | FJ411785 | T46 | M | genitalia | Maui | Halemau‘u Trail | 20.7524 | -156.2286 | 25 Jun 2000 | K. Magnacca |
|  | HYLHI259-08 | FJ411784 | T47 | M | right legs | Maui | Lau‘ulu Trail | 20.7314 | -156.1481 | 27 Jun 2000 | K. Magnacca |
|  | HYLHI260-08 | FJ411783 | T48 | M | right legs | Maui | Pu‘u Keokea | 20.6765 | -156.3171 | 20 May 2001 | K. Magnacca |
|  | HYLHI261-08 | FJ411782 | T119 | F | genitalia | Maui | Lau‘ulu Trail | 20.7314 | -156.1481 | 27 Jun 2000 | K. Magnacca |
| *Hylaeus nivicola* | HYLHI262-08 |  | T120n | M | 2 legs | Maui | Pu‘u Keokea | 20.6765 | -156.3171 | 20 May 2001 | K. Magnacca |
|  | HYLHI263-08 |  | T121n | M | 2 legs | Maui | Pu‘u Keokea | 20.6765 | -156.3171 | 20 May 2001 | K. Magnacca |
| *Hylaeus paradoxicus* | HYLHI264-08 |  | T125n | M | 2 legs | Hawai‘i | Pu‘u Wa‘awa‘a WS | 19.7362 | -155.8402 | 1 Aug 2002 | K. Magnacca |
| *Hylaeus rugulosus* | HYLHI265-08 | FJ411734 | T138n | F | thorax | Hawai‘i | Nāpau Trail | 19.3706 | -155.1536 | 14 Jul 2002 | K. Magnacca |
| *Hylaeus specularis* | HYLHI266-08 |  | T205n | M | 2 legs | Moloka‘i | above W. Kawela Stream | 21.1122 | -156.9071 | 27 Aug 2005 | K. Magnacca |
|  | HYLHI267-08 |  | C99n | M | genitalia | Kaua‘i | Nā Pali-Kona FR | 22.1434 | -159.6571 | 4 Jul 1999 | K. Magnacca |
|  | HYLHI268-08 |  | T131n | M | genitalia | Kaua‘i | ‘Awa‘awapuhi Trail | 22.1434 | -159.6571 | 5 Jul 2000 | K. Magnacca |
| *Hylaeus nivicola* | HYLHI269-08 | FJ411705 | T120p | M | 2 legs | Maui | Pu‘u Keokea | 20.6765 | -156.3171 | 20 May 2001 | K. Magnacca |
|  | HYLHI270-08 | FJ411704 | T121p | M | 2 legs | Maui | Pu‘u Keokea | 20.6765 | -156.3171 | 20 May 2001 | K. Magnacca |
| *Hylaeus paradoxicus* | HYLHI271-08 | FJ411717 | T125p | M | 2 legs | Hawai‘i | Pu‘u Wa‘awa‘a WS | 19.7362 | -155.8402 | 1 Aug 2002 | K. Magnacca |
| *Hylaeus rugulosus* | HYLHI272-08 | FJ411733 | T138p | F | thorax | Hawai‘i | Nāpau Trail | 19.3706 | -155.1536 | 14 Jul 2002 | K. Magnacca |
| *Hylaeus specularis* | HYLHI273-08 | FJ411745 | T205p | M | 2 legs | Moloka‘i | above W. Kawela Stream | 21.1122 | -156.9071 | 27 Aug 2005 | K. Magnacca |
|  | HYLHI274-08 | FJ411744 | C99p | M | genitalia | Kaua‘i | Nā Pali-Kona FR | 22.1434 | -159.6571 | 4 Jul 1999 | K. Magnacca |
|  | HYLHI275-08 | FJ411743 | T131p | M | genitalia | Kaua‘i | ‘Awa‘awapuhi Trail | 22.1434 | -159.6571 | 5 Jul 2000 | K. Magnacca |
|  |  |  |  |  |  |  |  |  |  |  |  |

Extraction codes beginning with C were done with the phenol-chloroform-isoamyl alcohol method, those beginning with T were performed with the Qiagen kit (see Methods); “n” and “p” appended to the extraction code denote numt and composite numt/coding sequences respectively. FR = Forest Reserve; HALE = Haleakalā National Park; HAVO = Hawai‘i Volcanoes National Park; KFU = Kona Forest Unit of Hakalau National Wildlife Refuge; NAR = Natural Area Reserve; NHP = National Historical Park; SP = State Park; WS = Wildlife Sanctuary.
